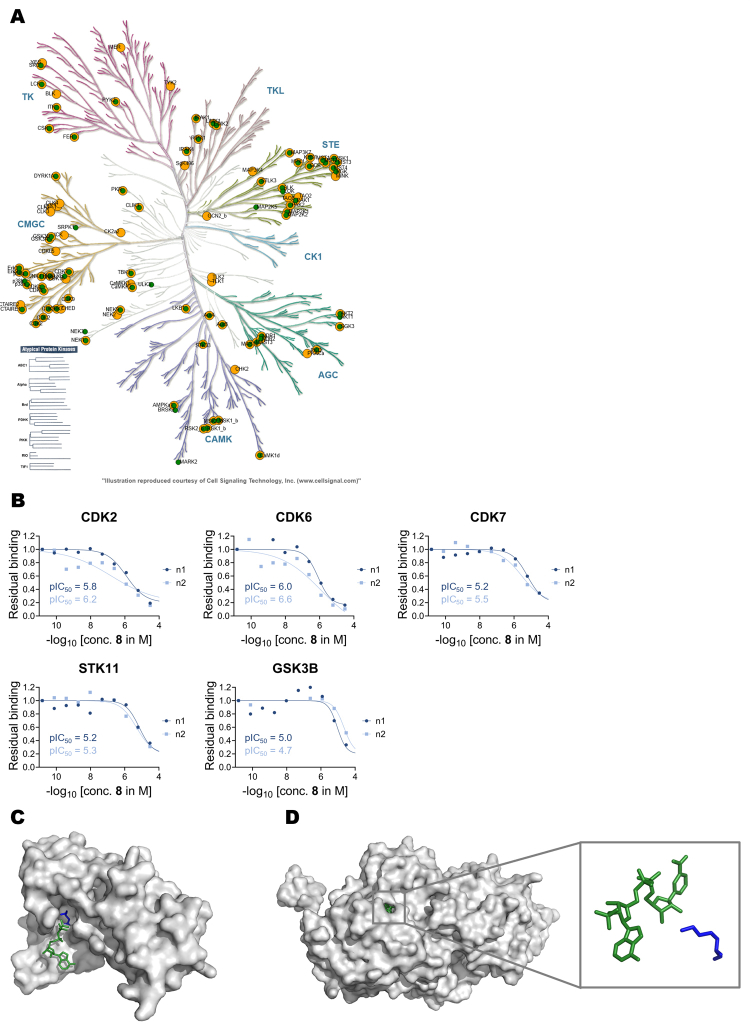# A Probe-Based Target Engagement Assay for Kinases in Live Cells

**DOI:** 10.1016/j.mcpro.2025.100963

**Published:** 2025-04-03

**Authors:** Ursula M. Glocker, Florian Braun, H. Christian Eberl, Marcus Bantscheff

**Affiliations:** 1 Cellzome, A GSK Company, Heidelberg, Germany; 2 Chemical Synthesis Core Facility, European Molecular Biology Laboratory, Heidelberg, Germany

**Keywords:** quantitative target engagement assay, covalent inhibitor, kinase, proteomics, mass spectrometry (MS), LC−MS/MS, TMT labeling

## Abstract

The efficacy and safety of kinase inhibitor drugs are largely influenced by their selectivity. Available profiling technologies are primarily based on overexpressed or endogenously expressed kinases in cell extracts. We compared kinase capture with the cell penetrant covalent probe XO44 to three derivatives and found that replacing the alkyne handle with a trans-cyclooctene group allowed the development of a more robust kinase capture and enrichment protocol. An intracellular chemoproteomics target profiling and engagement assay was devised by optimizing probe concentration and incubation time and using an isobaric mass tag–based strategy for relative quantification. Comparing intracellular kinase profiles of the marketed drug dasatinib and the tool compound dinaciclib with the lysate-based kinobeads assay revealed excellent agreement in rank-order of binding. Dinaciclib showed a systematic shift to higher IC_50_s, suggesting that intracellular cosubstrate concentrations, cell penetration of the compound, as well as kinase localization and complexes in live cells influence target profiles. Further, we show that sepiapterin reductase SPR and multidrug resistance protein 1 ABCC1 are off-targets of kinase inhibitor scaffolds with potential implications on efficacy and safety.

## Highlights

• Establishment of a cellular target engagement assay for kinases.
• More robust workflow enabled through IEDDA bioorthogonal reaction.
• Identification of common kinase inhibitor off-targets SPR and ABCC1.

## Graphical Abstract

**Figure:**
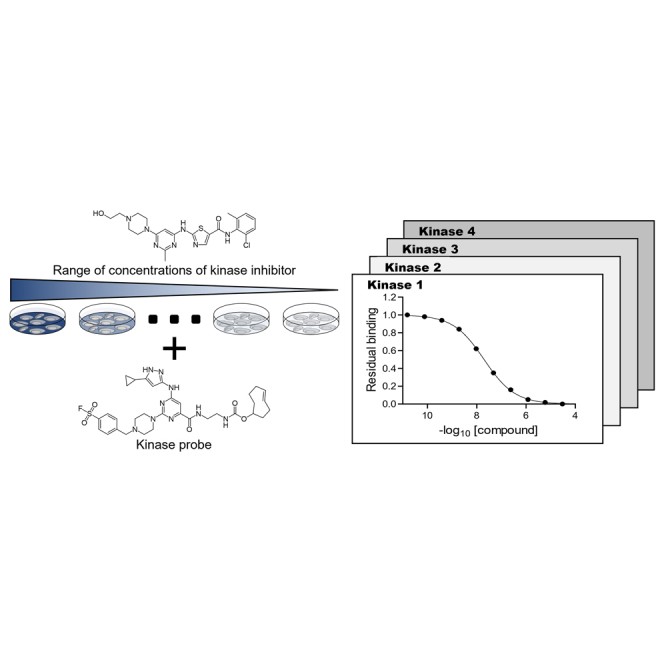

## In Brief

Available profiling technologies for protein kinases are primarily based on overexpressed or endogenously expressed kinases in cell extracts. We present and benchmark a robust chemoproteomics workflow for kinase inhibitor profiling in live cells using a trans-cyclooctene-modified analog of the promiscuous kinase inhibitor XO44.

Protein kinases (PKs) comprise one of the largest gene families with more than 500 members in human (1). Posttranslational modifications of proteins by PKs in response to extracellular signals are involved in a wide range of cellular processes (2), such as cell growth, metabolism, proliferation, differentiation, migration, effector functions, and cell death (3). Thus, the catalytic activity of PKs must be strictly regulated under physiological conditions (4). Dysregulated PK activity leads to the development of cancer and other diseases, making kinase inhibitors one of the most important drug classes of the 21st century (5). Most PK inhibitors were designed to target the highly conserved “druggable” ATP-binding pocket (6) and are directly competing for this pocket with high intracellular concentrations of the cosubstrate ATP (7). Consequently, kinase inhibitors are frequently not fully selective with polypharmacology, providing both opportunities for higher efficacy and increased therapeutic potential of a drug, as well as the risk of toxic side effects (8). This explains the need to thoroughly investigate and understand kinase inhibitor target- and off-target activities already in early-stage drug discovery to focus on safe and efficacious target profiles and to decrease risks for failure of drug candidates in later clinical phases (9). A plethora of kinase assays exists which can be performed at distinct levels of complexity, from recombinantly expressed purified proteins to endogenous kinases in cell extracts or even in live cells. An optimal assay would provide information on selectivity and potency which is highly predictive of the *in vivo* situation. Several lysate-based chemoproteomics methods have been developed, ranging from the usage of a single immobilized kinase inhibitor for affinity enrichment (10) *via* covalent ATP-biotin probes (“KiNativ”) (11, 12) to ATP (13, 14) or a set of promiscuous kinase inhibitors tethered to a solid support (“Kinobeads”) (15, 16, 17). Profiling of kinase inhibitors in live cells has been attempted with photoaffinity labeling probes (18); however, cell-permeable kinase probes that cover a substantial range of the human kinome were missing. This gap was addressed to some degree by the development of the cell penetrant and relatively promiscuous kinase inhibitor scaffold XO44 that was designed to react with the conserved catalytic lysine residue in PKs, present in all kinases except a small group of serine-threonine PKs that do not contain a lysine residue at this position, “WNKs” (with-no-lysine [K]) (19, 20). Initial data generated at two concentrations of the approved kinase inhibitor dasatinib suggested that binding preferences revealed with this cell-based approach may profoundly differ to published data that was based on lysate-based experiments. However, to our surprise, to this date, dose-dependent cellular binding studies and the description of a robust workflow have been lacking, thus limiting our ability to truly compare lysate- and cell-based chemoproteomics approaches. Here we describe a modified version of the XO44 probe that through inverse electron-demand Diels-Alder (IEDDA) reaction allows a more efficient and robust capturing of specifically bound kinases in cell-based experiments. This probe was then used to establish an optimized workflow for kinase profiling in dose-dependent studies and we demonstrate its application by characterizing intracellular target binding of the cancer drug dasatinib and the tool molecule dinaciclib.

## Experimental procedures

### Compounds

The compounds dasatinib (7), dinaciclib (8), EKI-785 (9), and QM385 (10) were purchased from APE x BIO Technology LLC. PF-6808472 (2) was purchased from Sigma-Aldrich (Cat. #PZ0306). Compounds 1, 3, 4, 5, 6 were synthesized in house. Detailed synthesis methods can be found in the supplemental methods section.

### Cell Culture

Jurkat clone E6.1 and HepG2 were derived from ATCC and cultured according to the manufacturer's instructions. K-562 and HEK293 were derived from DSMZ (German Collection of Microorganisms and Cell Cultures GmbH) and cultured according to the manufacturer’s instructions. Human placenta was supplied by ABS Inc.

### Kinobeads Pulldown

For a cell-based kinobeads assay, Jurkat cells were seeded at 10^6^ cells/ml and incubated with a kinase inhibitor at nine different concentrations (top concentration: 30 μM, followed by 1:4 dilution steps) or dimethylsulfoxide (DMSO) for 60 min at 37 °C. Cells were harvested at 400*g*, 4 °C, washed with PBS, and lysed in 3.5 pellet volumes of lysis buffer (50 mM Tris hydrochloride (Tris–HCl) pH 7.4, 5% glycerol, 1.5 mM magnesium dichloride (MgCl_2_), 20 mM sodium chloride (NaCl), 1 mM sodium orthovanadate (Na_3_VO_4_), 1% octylphenoxypolyethoxyethanol (IGEPAL CA-630), 1 mM DTT. Lysates were homogenized using a Bead ruptor machine (Omni Inc) using the following cycle settings: speed: 4 m/s, time: 0.10 s, cycles: 1. Benzonase was added to a final concentration of 250 U/ml and incubated for 1 h at 4 °C. NaCl and sodium fluoride (NaF) were added to reach final concentrations of 150 and 25 mM, respectively, followed by centrifugation for 10 min at 20,000*g* at 4 °C. The supernatant was centrifuged again for 1 h at 100,000*g*, 4 °C. Protein concentration was determined by Bradford assay (Bio-Rad); lysates were snap frozen in liquid nitrogen and stored at −80 °C. Lysates prepared from adherent cell lines were lysed in 2.25 pellet volumes lysis buffer, accordingly.

Cleared lysate aliquots (either Jurkat lysate or a mixture of HEK293, K562, HepG2, and placenta 1:1:1:1 to obtain broader kinome coverage) were subjected to kinobeads enrichment as described previously (17, 21). Briefly, lysate aliquots from nontreated cells were incubated with a kinase inhibitor at nine different concentrations (top concentration: 30 μM, followed by 1:5 dilution steps) or DMSO for 60 min at 4 °C, followed by incubation with kinobeads (1 mg protein: 7 μl dry kinobeads) for additional 60 min. Lysates from treated cells were directly subjected to the kinobeads matrix. Beads were thoroughly washed, and bound proteins were eluted with elution buffer (200 mM Tris HCl, 250 mM Tris Base, 20% glycerol, 4% SDS, 0.01% bromphenol blue, 50 mM DTT). Eluted proteins were captured on magnetic carboxylate–modified particles (Thermo Fisher Scientific, #45152105050250, #65152105050250) for 15 min at RT. Beads were washed 4× with 70% ethanol, and proteins were on-bead digested as described below.

### Generation of Blocked Neutravidin Beads

Neutravidin beads were modified to render Neutravidin resistant to lysyl endopeptidase (LysC) and trypsin as described before (22). Briefly, Neutravidin beads (Thermo Fisher Scientific, #29204) were washed in ≥10 bead volumes PBS-T (PBS + 0.1% Tween) buffer. Cyclohexanedione was added in a ratio of ∼1:25 (μl beads/mg cyclohexanedione) at pH 13 and incubated under overhead rotation at RT for 4 h. Then, beads were washed again with ≥10 bead volumes PBS-T. Dry beads were resuspended in 4% formaldehyde and 0.2 M sodium cyanoborohydride. The bead suspension was incubated again at RT with occasional vortexing for 2 h. 0.1 M Tris–HCl (pH 7.4) was added to the bead suspension, and beads were washed with ≥10 bead volumes PBS-T. After discarding the supernatant, a 1:1 (v/v) bead slurry was prepared in PBS-T and stored at 4 °C until subsequent use.

For the enrichment of trans-cyclooctene (TCO)-labeled proteins, biotin-tetrazine was loaded onto blocked neutravidin beads. Biotin-tetrazine (Jena Bioscience, # CLK-027-25) was added to a 1:1 blocked neutravidin bead slurry in PBS to reach 0.2 mM loading density and incubated overnight at 4 °C. Remaining free neutravidin was blocked with 1 mM biotin (Sigma-Aldrich, #B4501) for 30 min at 4 °C and thoroughly washed.

### Probe-Based Protein Profiling in Live Cells and Lysate

Jurkat cells were treated with test compounds for 60 min or 90 min either at 10 μM or DMSO (both in duplicate) or with nine different concentrations (30 μM top concentration and either 1:4 or 1:5 dilution steps) and DMSO, followed by addition of the probe (compound 2 or compound 3) at 1 μM for 30 min. Cells were harvested as described before.

For experiments with compound 2 (alkyne click handle), frozen cell pellets were lysed with three pellet volumes of lysis buffer (50 mM 4-(2-Hydroxyethyl)piperazine-1-ethane-sulfonic acid (Hepes) pH 8, 0.5% IGEPAL CA-630, 20 mM NaCl, 1.5 mM MgCl_2_, protease inhibitor cocktail (Sigma-Aldrich, #P8340) 1:100). Benzonase was added to a final concentration of 250 U/ml and incubated for 1h at 4 °C. NaCl, IGEPAL CA-630, SDS, and sodium deoxycholate concentrations were adjusted to 150 mM, 1%, 0.1%, and 0.5%, respectively. Lysates were subjected to ultracentrifugation at 100,000 g at 4 °C for 1 h.

For lysate-based protein profiling with compound 2, cells were harvested and lysed prior to incubation with test compounds for 60 min at 10 μM or DMSO (both in duplicate), 4 °C, followed by addition of the probe (compound 2) at 1 μM for 30 min at 4 °C. Detergent concentrations were adjusted afterward.

To each protein concentration–adjusted cleared lysate, a copper-click mixture (final concentrations of 100 μM Biotin-PEG3-azide, 1 mM CuSO_4_, 1 mM tris(2-carboxyethyl)phosphine, and 100 μM 2-(4-((bis((1-(tert-butyl)-1H-1,2,3-triazol-4-yl)methyl)amino)methyl)-1H-1,2,3-triazol-1-yl)acetic acid) was added and incubated for 1 h at RT shaking at 1000 rpm. The click reaction was quenched by adding 10 mM EDTA. Proteins were precipitated with acetone (final concentration 80%) overnight at −20 °C and precipitates were washed twice with 80% acetone. Air-dried pellets were resolubilized in 0.2% SDS in 50 mM Hepes, pH 8.0, and enrichment was performed on protease-resistant Neutravidin beads (ratio of 1 mg protein: 35 μl dry beads) for 2 h at RT. After protein binding, beads were extensively washed: 4× wash buffer 1 (50 mM Hepes pH 8.5, 400 mM NaCl, 0.5% SDS), 8× wash buffer 2 (50 mM Hepes pH 8.5, 400 mM NaCl), 8× wash buffer 3 (50 mM Hepes pH 8.5, 2 M Urea), 4 × 50 mM Hepes pH 8.5.

For experiments with compound 3 (TCO handle), (treated or untreated) cells were lysed in three pellet volumes lysis buffer (50 mM Tris–HCl pH 7.4, 5% glycerol, 1.5 mM MgCl_2_, 20 mM NaCl, 1 mM Na_3_VO_4_, 28 nM aprotinin, 2.2 μM bestatin, 5.5 μM leupeptin, 80 nM pepstatin A, 0.5 μM phosphoramidon, 1 mM DTT). Lysates were homogenized using a bead ruptor machine (Omni Inc.) using the above shown cycle settings. Benzonase was added to a final concentration of 250 U/ml and incubated for 1 h at 4 °C. NaCl and NaF were added to reach final concentrations of 150 and 25 mM, respectively.

For lysate-based protein profiling with compound 3, cells were harvested and lysed prior to incubation with test compounds for 60 min at 10 μM or DMSO (both in duplicate), 4 °C, followed by addition of the probe (compound 3) at 1 μM for 30 min at 4 °C.

Lysate concentrations were adjusted to 1.1 ml with 1× drug pulldown (DP) buffer (50 mM Tris–HCl (pH 7.4), 5% glycerol, 1.5 mM MgCl_2_, 150 mM NaCl, 1 mM Na_3_VO_4_, 25 mM NaF) such that all lysates belonging to one experiment have the same final protein concentration. SDS was added to a final concentration of 0.5%, followed by centrifugation at 20,000g for 20 min. One milliliter of lysate was added to 35 μl dry tetrazine-loaded neutravidin beads and incubated for 1 h at 4 °C. After protein binding, beads were extensively washed: 5x 1x DP buffer, 0.4% IGEPAL CA-630, 0.5% SDS, 3x 1x DP buffer, 0.2% IGEPAL, 0.5% SDS, elution buffer for 30 min at 50 °C, 4× wash buffer 1 (50 mM Hepes pH 8.5, 400 mM NaCl, 0.5% SDS), 8× wash buffer 2 (50 mM Hepes pH 8.5, 400 mM NaCl), 8× wash buffer 3 (50 mM Hepes pH 8.5, 2 M Urea), 4 × 50 mM Hepes pH 8.5.

### MS Sample Preparation and LC-MS/MS Analysis

Digest buffer (5 mM tris(2-carboxyethyl)phosphine, 15 mM chloroacetamide, 50 mM Hepes pH 8.5, 0.0048 μg/μl LysC, 0.0048 μg/μl trypsin) was added for overnight digest at room temperature. Peptides were collected, and elution was completed by washing the beads with 50 mM Hepes, pH 8.5 or HPLC-grade water (for kinobeads experiments).

Peptide mixtures were labeled with the respective 10-plex tandem mass tag (TMT) label (Thermo Scientific), enabling relative quantification of up to 10 conditions in a single experiment for 1 h at RT. Nonreacted TMT was quenched with 2.5% hydroxylamine (NH_2_OH) at RT for 15 min. Peptide mixtures corresponding to one TMT experiment were pooled and subjected to C18-strong cation exchange (C18SCX) sample cleanup to remove residual detergent, salts, and/or TMT reagent. C18SCX material was equilibrated with methanol, followed by centrifugation (2000 g, 2 min) and addition of 2% acetonitrile (ACN)/0.5% TFA. Dried peptides were resuspended in 6% TFA and were added to the equilibrated C18SCX material. After addition of 100 μl 0.5% TFA/2% ACN which was followed by centrifugation (2000 g, 2 min), peptides were eluted onto the SCX material by adding 0.5% TFA/60% ACN which was followed by centrifugation at 2000*g* for 2 min. Cleaned peptides were eluted with 100 μl 5% NH_3_/80% ACN. Peptide solutions were dried in vacuo. Mass spectrometric analysis was performed as described previously (17). Briefly, lyophilized samples were resuspended in 0.05% TFA in water, and 30% of each sample were injected into an Ultimate3000 nanoRLSC (Dionex) coupled to an Orbitrap Exploris 480 (Thermo Fisher Scientific) or an Orbitrap Eclipse (Thermo Fisher Scientific) mass spectrometer. Peptides were separated on custom-made 50 cm × 100 μm (inner diameter) reversed-phase columns (C18, 1.9 μm, Reprosil-Pur, Dr Maisch) at 55 °C. Gradient elution was performed from 2% ACN to 36% ACN in 0.1% formic acid and 3.5% DMSO over 200 min at a flow rate of 350 nl/min. Eluting peptides were online injected into the mass spectrometer operating with a data-dependent top 10 method. Mass spectra were acquired from 375 to 1200 m/z by using 60,000 resolution and an ion target of 3 × 10^6^ for MS1 scans including charge states 2 to 4 (Orbitrap Exploris 480) or 2 to 6 (Orbitrap Eclipse). Higher energy collisional dissociation (HCD) scans were performed with 33% normalized collision energy at 30,000 resolution (Orbitrap Exploris 480) or with 38% normalized collision energy at 15,000 resolution (Orbitrap Eclipse); first mass for MS2 scans was set to 100 m/z, isolation window was set to 0.7 m/z, and the ion target for MS2 was set to 2 × 10^5^ so as to avoid coalescence (23). The instruments were operated with Tune 2.4 and Xcalibur 3.0 build 63.

### Peptide and Protein Identification and Quantification

Raw data were processed using an in-house pipeline based on the isobar quant package (24). Mascot 2.5 (Matrix Science) was used for protein identification. Enzyme specificity was set to trypsin with up to three missed cleavages. Peptides of charges 2^+^ and 3^+^ were included. In a first search, 30 ppm peptide precursor mass and 30 mDa (HCD) mass tolerance for fragment ions was used for recalibration according to Cox *et al*. (25), followed by search using a 10 ppm mass tolerance for peptide precursors and 20 mDa (HCD) mass tolerance for fragment ions. The search database consisted of a customized version of the SwissProt sequence database (SwissProt Human, release December 2018, 42,423 sequences) combined with a decoy version of this database created using scripts supplied by Matrix Science. Carbamidomethylation of cysteine residues and TMT modification of lysine residues were set as fixed modifications. Methionine oxidation, N-terminal acetylation of proteins, and TMT modification of peptide N-termini were set as variable modifications. We accepted protein identifications as follows: (i) for single spectrum-to-sequence assignments, we required this assignment to be the best match, a minimum mascot score of 31 and a 10 × difference of this assignment over the next best assignment. Based on these criteria, the decoy search results indicated <1% false discovery rate. (ii) For multiple spectrum-to-sequence assignments and using the same parameters, the decoy search results indicate <0.1% false discovery rate. Reporter ion intensities were read from raw data and multiplied with ion accumulation times (in milliseconds) so as to yield a measure proportional to the number of ions; this measure is referred to as ion area (26). Spectra matching to peptides were filtered according to the following criteria: mascot ion score >15, signal-to-background of the precursor ion >4, and signal-to-interference >0.5 (27). Fold changes (FCs) were corrected for isotope purity as described and adjusted for interference caused by co-eluting nearly isobaric peaks as estimated by the signal-to-interference measure (28). Protein quantification was derived from individual spectra matching to distinct peptides by using a sum-based bootstrap algorithm; 95% confidence intervals were calculated for all protein FCs that were quantified with more than three spectra. Only proteins quantified with more than one unique peptide (qupm) were considered for downstream analysis. The mass spectrometry proteomics data have been deposited to the ProteomeXchange Consortium *via* the PRIDE (29) partner repository with the dataset identifier PXD047949.

### Experimental Design and Statistical Rationale

Data analysis and visualizations were performed in R (version 4.1). Kinome tree figures were plotted using KinMap_beta_ (KinHub (kinhub.org/kinmap/)). Dose-response curves that are shown in this paper were performed in GraphPad Prism 9 (version 9.3.1 (471)), as well as the time-dependent labeling of proteins. Protein structures were visualized using PyMol (Schrodinger, LLC, version 2.3.2). All experiments were performed using a TMT-labeling strategy. All experiments were performed in biological duplicates. The target engagement assay with dasatinib was performed in five biological replicates.

For each TMT experiment, one biological replicate of compound or (kinase) inhibitor treatment, probe titration, or time titration treatment were combined into one multiplexed TMT10 experiment, except for the single concentration competition experiments, where biological duplicates were combined in one TMT4 experiment. Corresponding duplicate experiments were merged for further analysis. All identified proteins were filtered at qupm >1 and potential contaminants were removed. Differences in specific kinase binding with the kinobeads assay were analyzed by two-tailed *t* test. For target engagement assays, only “specific binders” (as determined by single concentration competition) were included in the analysis. Data was filtered for the specific binders and normalized over the median relative FC per TMT channel of specific binders. Proteins with “bad” curve quality after normalization were removed. Dose-response curves were fitted using R (http://www.r-project.org/) and the 4-parameter logistic model of the drc package (http://www.bioassay.dk). Data was prefiltered to obtain competed proteins. For these proteins, a parameter was fixed for the bottom of the curve to be either set to 0.2 if the protein was not fully competed or to the lowest FC obtained. With this prefiltered data and setting (some of) the curve-fitting parameters, the algorithm fits the FCs to the dose response model. The algorithm first tries a regular fitting and then tries to calculate coefficients. From these coefficients a log confidence delta is calculated. If no dose-response curve could be fitted, the QC-parameter was set to bad as well as for the case that all datapoints were competed. Further, if the log confidence delta was >0.45, the QC-parameter was set to bad.

## Results

**Fig. 1:**
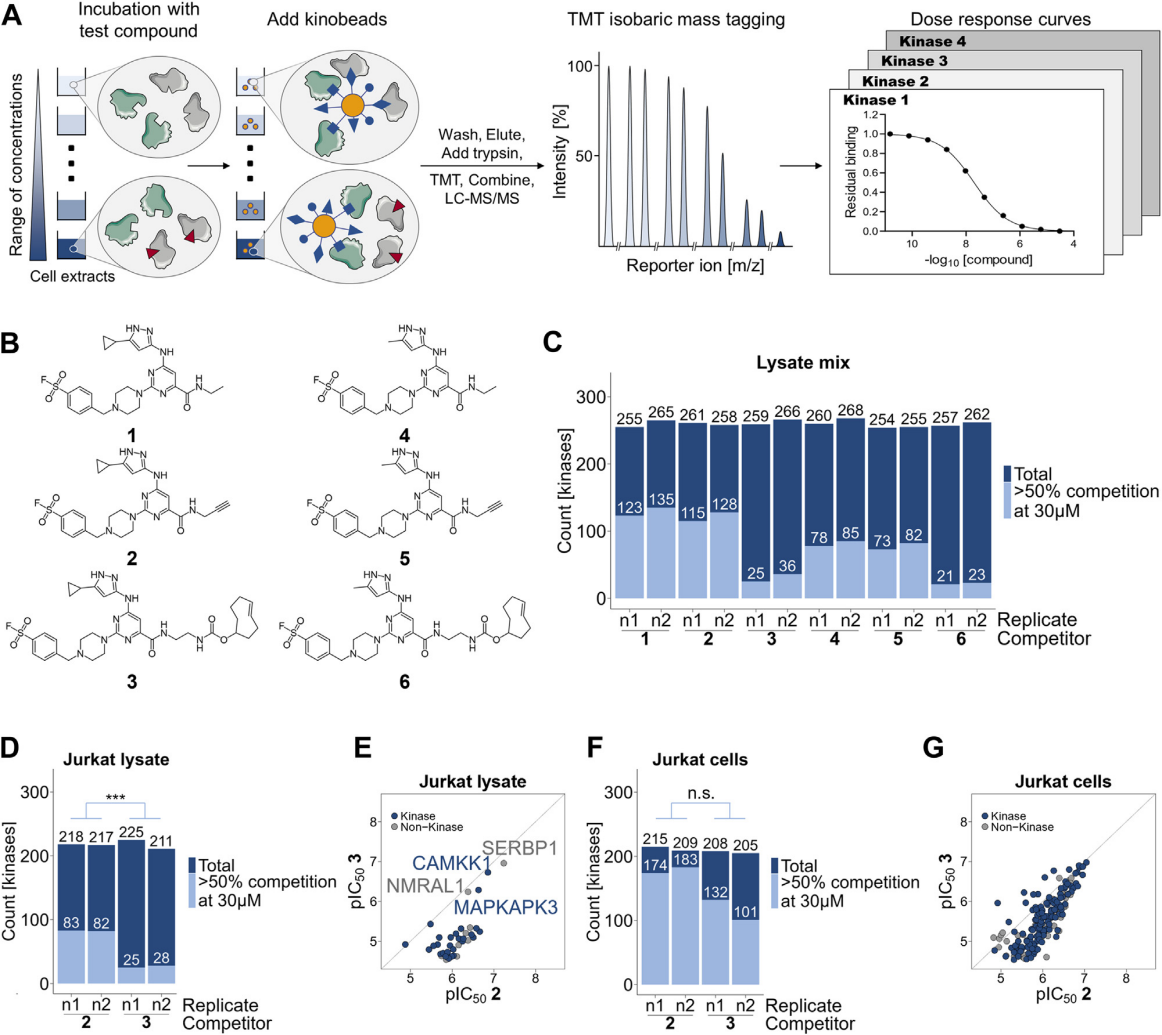
**Characterization of probes using chemoproteomics selectivity profiling with kinobeads.***A*, schematic representation of the kinobeads workflow. Cell extracts or cells are treated with a compound (*red triangle*) using a range of concentrations or vehicle control (*left*). Protein mixtures are schematically depicted in *gray circles*; proteins depicted in *gray* display target kinases and proteins depicted in *green* display nontargets. Protein kinases that have bound a compound in the ATP-binding site are unavailable for binding to kinobeads that are comprised of a set of unspecific ATP-competitive kinase inhibitors (*yellow circles* with various *blue* shapes). Unbound proteins are washed off. Proteins bound to kinobeads are eluted and subsequently digested with trypsin and LysC. Each peptide pool is labeled with a different variant of the TMT reagent, then peptide mixtures are combined and analyzed by MS. Each peptide gives rise to characteristic TMT reporter signals indicative of the compound concentration used. For each peptide detected, the decrease of signal intensity compared to the vehicle control reflects competition by the “free” compound for its target (*right*). *B*, sulfonyl fluoride–containing compounds derived from a pyrimidine 2-aminopyrazole kinase-recognition scaffold (XO44) used for characterization with kinobeads. *C*, kinobeads analysis using a mixture of cell extracts (HepG2 Princen, K-562, Hek293, placenta) for compounds 1 to 6 (n = 2). Depiction of total number of quantified kinases and subset of competed kinases (>50% competition at 30 μM) per replicate. *D*, kinobeads analysis using Jurkat cellular extract for compounds 2 and 3 (n = 2). Depiction of the total number of quantified kinases and subset of competed kinases (>50% competition at 30 μM) per replicate. ∗∗∗, *p* = 0.0008, two-tailed *t* test. *E*, comparison of the binding strengths of competed proteins (>50% competition at 30 μM determined in D) of compounds 2 and 3 in Jurkat cellular extracts. Diagonal line represents 1:1 line. *F*, kinobeads analysis after treatment of Jurkat cells with compounds 2 and 3 for 60 min (n = 2). Depiction of the total number of quantified kinases and subset of competed kinases (>50% competition at 30 μM) per replicate. n.s.: *p* = 0.06, two-tailed *t* test. *G*, comparison of the binding strengths of competed proteins (>50% competition at 30 μM determined in F) of compounds 2 and 3 in Jurkat cells. Diagonal line represents 1:1 line.

Using the scaffold of the previously described broad-spectrum kinase inhibitor XO44 as template (19), we synthesized a small set of covalent kinase-targeting tools (Fig. 1*B*) consisting of an XO44 analog without click-handle as control molecule (1), the original XO44 molecule containing an alkyne click handle (2), and molecule 3, where the click handle was replaced with a TCO moiety that enables the faster and more efficient bioorthogonal IEDDA reaction (30) for subsequent affinity enrichment. In addition, we generated a similar set of molecules where the cyclopropyl residue was replaced with a smaller methyl group (4–6).

We assessed the specific kinase binding of the probe set with a kinobeads assay, an established lysate-based chemoproteomics selectivity profiling assay (Fig. 1*A*) (17). Kinobeads consist of a small set of immobilized promiscuous ATP-competitive kinase inhibitors, and more than 250 PKs were captured from a mixture of cell extracts (Fig. 1*C* and supplemental Table S1). The subset of these kinases that was specifically bound to any of the probes was determined in competitive binding experiments where we required at least 50% reduction in kinobeads binding at 30 μM probe concentration.

XO44 and the analog without click handle 1 affected binding to kinobeads of about half of the kinases that were captured under these conditions. Exchanging the cyclopropyl group with the smaller methyl group reduced the number of competed kinases by approximately 30% when comparing compounds without bioorthogonal handle or alkyne-containing compounds (Fig. 1*C*, compound 1–4, 2–5), and replacing the alkyne handle with a TCO moiety (compounds 3 and 6) substantially impaired kinase binding (Fig. 1*C*).

**Figure S1:**
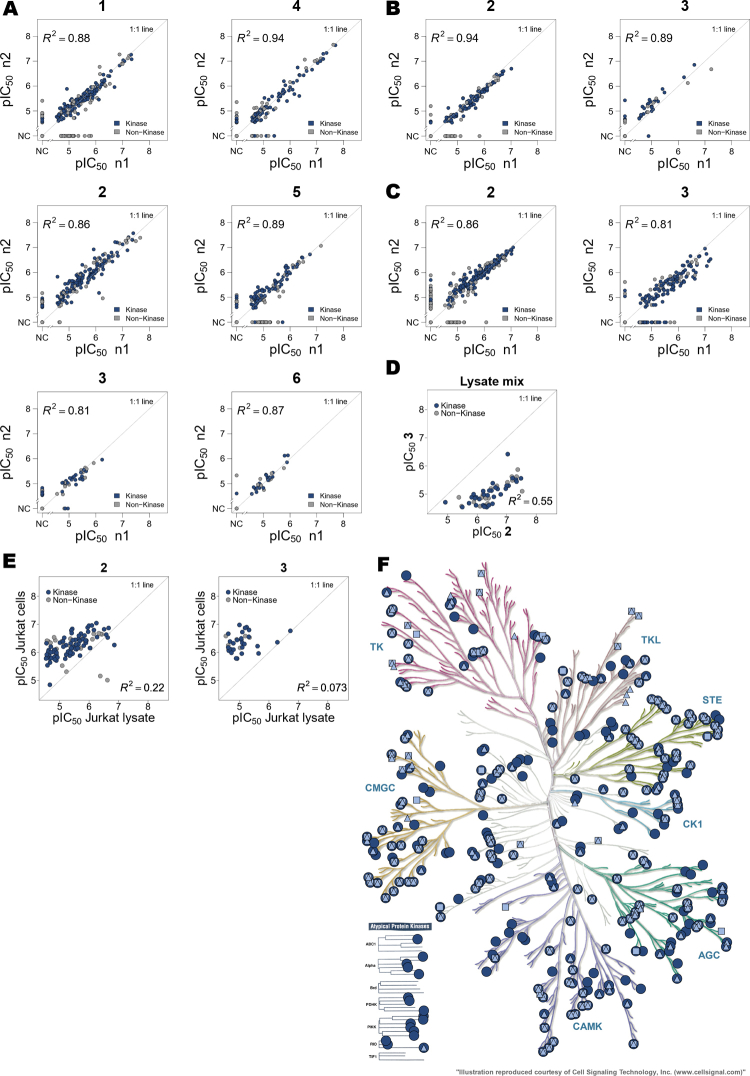


Next, we determined the kinase-binding ability of compounds 2 and 3 in live cells and cell extracts derived from the Jurkat T-cell model by using the kinobeads assay. Jurkat cells express the majority of the kinases we had found to be specifically affected by probes 2 or 3 in the lysate mix kinobeads experiments (Fig. 1, supplemental Fig. S1 and supplemental Table S2).

Of the 200 PKs captured with kinobeads from Jurkat lysate, binding of more than 80 kinases was reduced by more than 50% at 30 μM 2, whereas 3 only affected kinobeads binding of approximately 20 kinases confirming our previous observation that replacement of the alkyne click handle for a TCO group impaired kinase capture from cell extracts (Fig. 1, *D* and *E* and supplemental Fig. S1*B*). Interestingly, some proteins, such as the kinases CAMKK1 and MAPKAPK3, showed no lower binding strength upon binding to 3 (Fig. 1*E*). However, when live Jurkat T-cells were incubated for 60 min with these probes before cell lysis and kinase capture with kinobeads, both probes showed increased kinase binding compared to experiments performed with cell extracts (Fig. 1*F* and supplemental Fig. S1, *B* and *C*), and the difference in binding strength of 3 *versus* 2 was less pronounced (Fig. 1, *F* and *G* and supplemental Fig. S1, *C* and *E*).

**Fig. 2:**
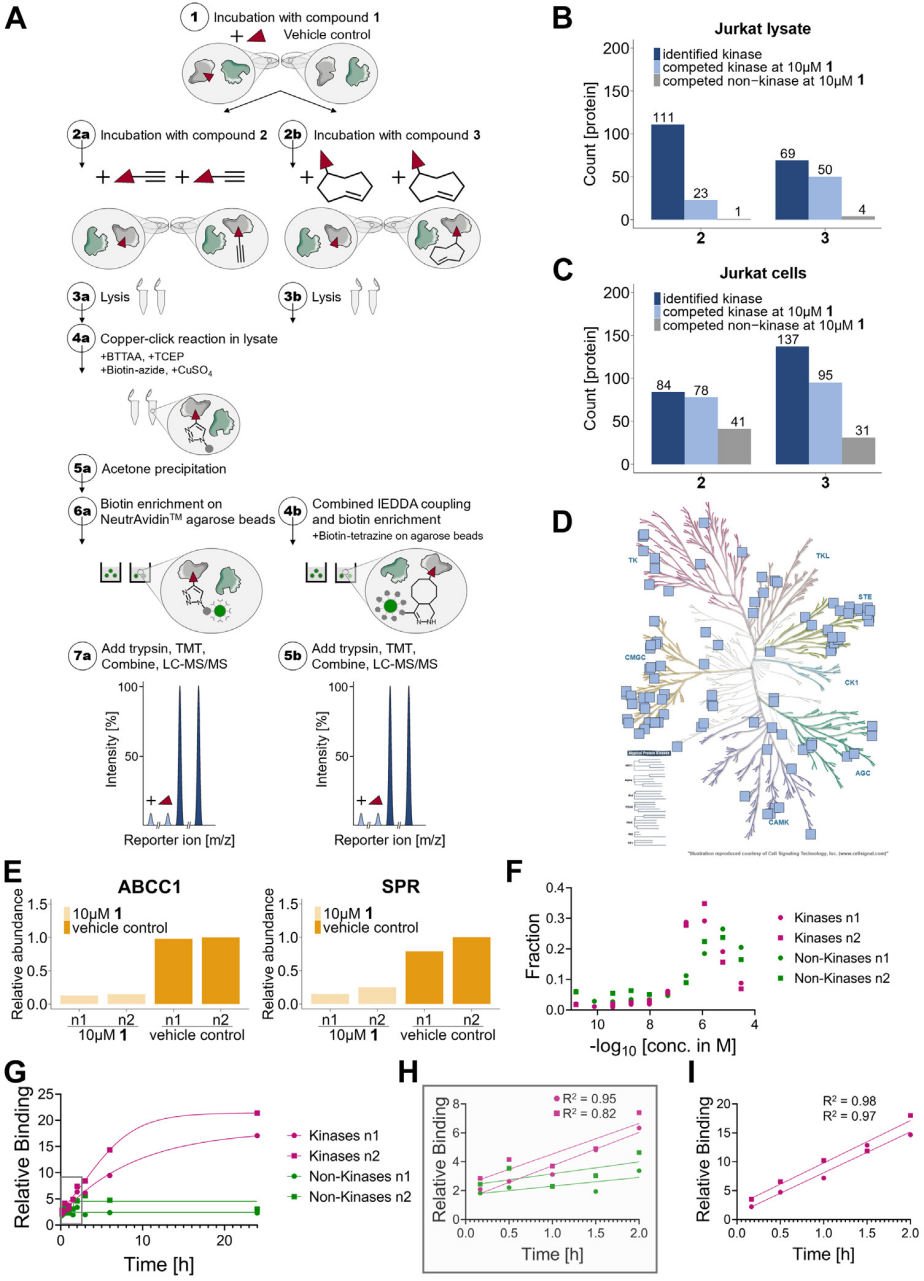
**Target engagement assay using promiscuous kinase probes.***A*, schematic of the different workflows for copper-click or inverse electron-demand Diels-Alder (IEDDA) chemistry-based enrichment experiments. Cells are treated with 1 (*red triangle*) or vehicle control (①), followed by subsequent incubation with 2 (*red triangle* containing an alkyne handle, ②a) or 3 (*red triangle* containing a trans-cyclooctene (TCO) handle, ②b), competing for the kinases’ ATP-binding site. When being pre-incubated with compound 1, targeted kinases are less available for binding to the probes 2 or 3. Following lysis (③a+b), proteins that are covalently bound to compound 2 undergo copper-click reaction in lysate to click on biotin (④a). Copper(II) sulfate (CuSO_4_) is reduced to copper(I) with the reducing agent tris(2-carboxyethyl)phosphine (TCEP). BTTAA is used as the catalyst. Subsequently, proteins are acetone precipitated (⑤a) and resolubilized, followed by capturing on NeutrAvidin agarose beads (⑥a). Proteins that are covalently bound to compound 3 are captured on biotin-tetrazine–modified NeutrAvidin agarose beads by IEDDA chemistry (④b). Subsequently, unbound proteins are washed off and bound proteins are digested with trypsin and LysC. Each peptide pool is labeled with a different variant of the TMT reagent (not shown). All peptide mixtures are combined and analyzed by MS. Each peptide gives rise to characteristic TMT reporter signals (scaled to 100%) indicative of the compound concentration used (⑦a+⑤b). *B*, profiling in Jurkat cellular extracts. Lysates are pre-incubated for 60 min with 10 μM compound 1 or vehicle control followed by incubation with 1 μM compound 2 or 3 for 30 min. Depicted are all robustly identified kinases (quantified peptide matches ≥2), and the subset of kinases or nonkinase proteins that are competed by 1 from the probes. Proteins are defined as competed when the relative abundance of both TMT channels pre-incubated with 1 compared to the first DMSO channel is < 0.5 and the relative abundance of the second DMSO channel to the first DMSO channel is > 0.5. *C*, same as in (*B*) but using live Jurkat cells. *D*, kinome tree representation of specifically captured and competed kinases by compound 3 in Jurkat cells (95 kinases, as shown in (*C*)). *E*, individual protein quantification from profiling in Jurkat cells (*C*) for ABCC1 and SPR—two nonkinase proteins specifically captured by compound 3. *F*, concentration-dependent capturing of kinases and nonkinases in Jurkat cells with 3 (n = 2). For each experiment, the cumulative intensity for all kinases or nonkinases for each TMT channel (corresponding to one concentration of 3) was divided by the total intensity over all channels for all kinases or nonkinases. The sum of all points per experiment per group (kinase or nonkinase) is 1 and the graph displays the relative concentration-dependent behavior of specifically (kinases) and unspecifically (nonkinase) captured proteins by 3. *G*, time-dependent capturing of kinases and nonkinases in Jurkat cells by 1 μM compound 3. Relative intensity to a vehicle control at 10 min for all kinases (*purple*) and all nonkinases (*green*) are shown (n = 2). *H*, zoom in on 0 h – 2 h of (*G*) to demonstrate linear time-dependent increase for all kinases within the first 2 h. *p*-values of the linear fits are *p* = 0.0041 (n1) and *p* = 0.035 (n2). *I*, zoom in on 0 h – 2 h of (*G*) representing the average relative binding of AURKA, PRKAA1, NEK9, STK35, and BLK. These kinases were most potently bound by 3 in the cellular kinobeads experiments. Linearity of binding indicates the absence of saturation and hence little impact on the interaction of test compounds with these kinases. *p*-values of the linear fits are *p* = 0.0015 (n1) and *p* = 0.0023 (n2).

Having established the kinase-binding potential of 2 and 3 using the kinobeads assay, we evaluated their use as kinase enrichment tools. Jurkat cells or cell extracts were incubated with 10 μM 1 or vehicle control for 60 min followed by the addition of 1 μM 2 or 3 for 30 min. Covalently modified proteins were enriched using either (2) copper-catalyzed azide–alkyne cycloaddition to biotin-azide in cell extracts followed by biotin enrichment on NeutrAvidin beads or (3) by directly enriching TCO-modified proteins on NeutrAvidin beads loaded with biotin-tetrazine. Bead-captured proteins were trypsinized, labeled with TMT reagents, and analyzed by quantitative mass spectrometry (Fig. 2*A*). In Jurkat cellular extracts, 2 captured 133 PKs of which 111 were robustly (with more than one quantifiable peptide from both replicates) captured, but only 23 of which showed substantially reduced binding in the presence of 10 μM 1. In contrast, 86 PKs were captured in total and 69 robustly with 3 of which 50 were competed by 1 (Fig. 2*B* and supplemental Table S3). This trend was even stronger when 2 or 3 were added to live Jurkat cells. With the alkyne-containing 2, 108 kinases were captured, 84 of which were robustly captured and 78 were competed, while with 3, 171 kinases were captured, of which 137 kinases of these were robustly captured and 95 were competed with 10 μM 1 (Fig. 2*C*). Of the 84 and 137 captured kinases with probes 2 and 3, respectively, 75 are captured with both probes, and of the 78 and 95 competed kinases, 68 overlap (supplemental Fig. S4*A*). The 95 kinases are distributed over the complete kinome tree, with some biases towards the CGMC and STE branches, while kinases located on the branch of CK1 or receptor tyrosine kinases are not captured (Fig. 2*D*). Although 3 was less effective in competing kinases in the kinobeads experiments, the more efficient IEDDA reaction for enrichment that requires fewer manual handling steps contributed to the improved kinase enrichment compared to 2. In addition to kinases, 41 and 31 nonkinase proteins were specifically and robustly captured by 2 and 3, respectively. Among those proteins are the translocase multidrug resistance protein 1 (ABCC1) and the oxidoreductase sepiapterin reductase (SPR) (Fig. 2*E*).

**Figure S2:**
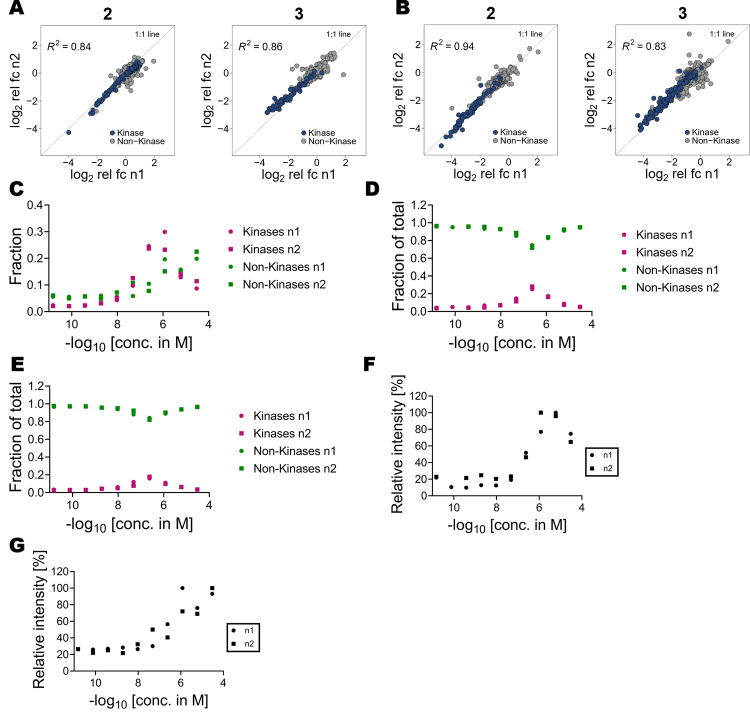


Next, we focused on the use of 3 to quantitatively determine cellular target engagement of the (covalent or noncovalent) test compounds. In an ideal case, only a small proportion of each kinase is modified by the probe to minimize interference with the interaction of kinases with compounds of interest (see Supplemental Note for details) (12, 31). First, we determined relative amounts of specific binding—approximated by kinases, and unspecific binding—approximated by other proteins, as a function of probe concentration. Jurkat T-cells were incubated for 30 min with 3 covering a concentration range from 80 pM to 30 μM followed by cell lysis, enrichment of labeled proteins, and quantitative proteomics analysis. The summed-up TMT reporter ion abundances of kinase peptides increased up to a probe concentration of 1.2 μM but were reduced at higher concentrations of 3. In contrast, ion signal abundances of other proteins increased up to 6 μM probe concentration but significantly dropped at even higher concentrations (Fig. 2*F*, supplemental Fig. S2*D* and supplemental Table S4). Consistent with this, similar effects were observed when the experiment was performed in Jurkat cellular extract (supplemental Fig. S2, *C* and *E*). A notable exception however was that the intensity drop for kinases and nonkinase proteins at the highest concentration of 3 was only observed in experiments in Jurkat cells (Fig. 2*F* and supplemental Fig. S2, *C*–*G*).

In a next step, we investigated the time-dependent labeling of proteins by 1 μM 3 in live Jurkat T-cells over eight time points ranging from 10 min to 24 h. We observed a time-dependent increase in the cumulative ion abundance of kinase peptides with only a slight increase in the capturing of other proteins (Fig. 2*G*). This data confirms compound stability in the cellular setting over a long period of time. The relative increase of the amount of kinases captured was linear within the first 2 h (Fig. 2*H*) even for those kinases most potently bound by 3 (Fig. 2*I*). These results suggest that using 3 at 1 μM for probing noncovalent kinase inhibitor target occupancy at incubation times less than 2 h will have a negligible influence on the equilibrium as the probe only captures a small fraction of the available kinase pool (12). Hence, we decided to use compound 3 at 1 μM for 30 min in all subsequent experiments. This time point allows for robust experimentation while still being on the lower end of the linear signal increase.

**Fig. 3:**
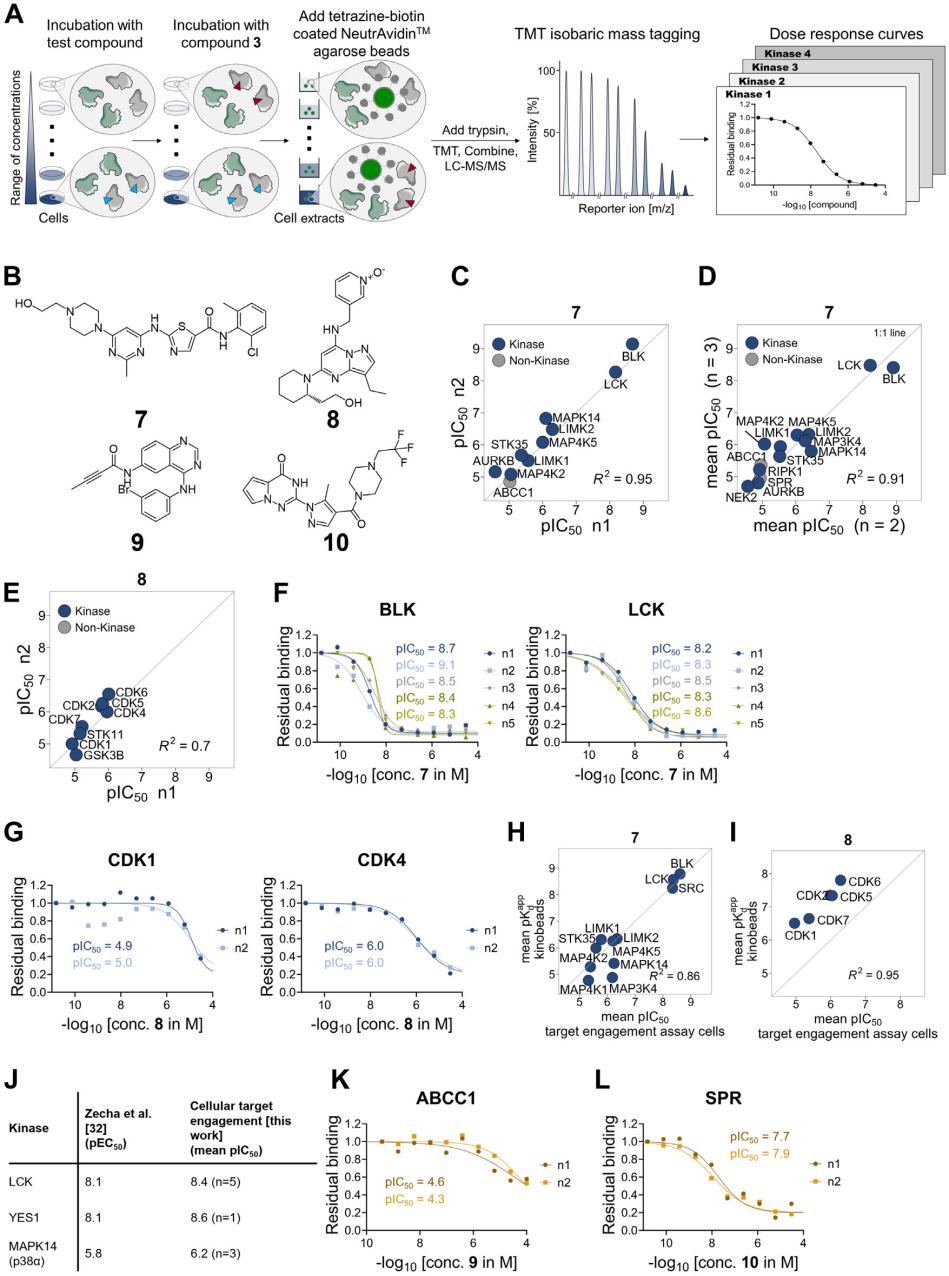
**Kinase target engagement assay using compound 3.***A*, schematic of the target engagement assay in live cells using compound 3. Cells are treated with a test compound (*light blue triangle*) using a range of concentrations as well as a vehicle control (*left*). After addition of 3 (*red triangle*) to all samples, compound 3 covalently reacts with its targets; targets engaged by the test compound cannot be labeled by compound 3. Proteins covalently modified by compound 3 are captured on biotin-tetrazine–modified NeutrAvidin agarose beads by IEDDA chemistry. Bead occupancy for targets of the test compound decreases reciprocally to test compound concentration (*middle*). Unbound proteins are washed off. Bound proteins are digested with trypsin and LysC. Each peptide pool is labeled with a different variant of the TMT reagent (not shown). All peptide mixtures are combined and analyzed by MS. Each peptide gives rise to characteristic TMT reporter signals (scaled to 100%) indicative of the compound concentration used (*middle right* panel). For each protein detected, the decrease of signal intensity compared to the vehicle control reflects competition by the “free” compound for its target (*right*). *B*, chemical structures of the three kinase inhibitors dasatinib (7), dinaciclib (8), and EKI-785 (9), as well as the SPR inhibitor QM385 (10) used for competition experiments. *C*, scatterplot illustrating the reproducibility of the target engagement assay in live Jurkat cells testing kinase inhibitor 7. Comparison of determined pIC50s in replicates one and two (all possible comparisons between each of five replicates are shown in supplementary Fig. S3*A*). Diagonal line represents 1:1 line. R2 is derived from a linear fit without predefined intercept. *D*, comparison of mean pIC50s from target engagement assays in live Jurkat cells testing kinase inhibitor 7. Triplicates (n = 3) were compared to fully independent replicates (n = 2) performed a year earlier. Diagonal line represents 1:1 line. R2 is derived from a linear fit without predefined intercept. *E*, scatterplot illustrating the reproducibility of the target engagement assay in live Jurkat cells testing kinase inhibitor 8. Comparison of determined pIC50s between replicates (n = 2). Diagonal line represents 1:1 line. R2 is derived from a linear fit without predefined intercept. *F*, exemplary residual-binding curves of the selected competed kinases BLK and LCK using test compound 7 (dasatinib) (Fig. 3, *C* and *D*) (n = 5). *G*, exemplary residual-binding curves of the selected competed kinases CDK1 and CDK4 using test compound 8 (dinaciclib) (Fig. 3*E*) (n = 2). *H*, comparison of mean pIC50 values determined by the cellular target engagement assay in Jurkat cells (n = 5) with mean apparent dissociation constants (pKdapp) determined by kinobeads in Jurkat lysate (n = 2) for the kinase inhibitor 7. Diagonal line represents 1:1 line. R2 is derived from a linear fit without predefined intercept. *I*, comparison of mean pIC50 values determined by the cellular target engagement assay in Jurkat cells with mean apparent dissociation constants (pKdapp) determined by kinobeads in Jurkat lysate (n = 2) for the kinase inhibitor 8. Diagonal line represents 1:1 line. R2 is derived from a linear fit without predefined intercept. *J*, comparison of pEC50 values determined in a phosphoproteomics study by Zecha *et al*. Thirty two to pIC50 values generated using the cellular target engagement assay described here. The number of replicates with coverage of these kinases is shown in brackets. *K*, target engagement assay in live Jurkat cells with test compound 9. Dose-response curve for ABCC1 (n = 2). *L*, target engagement assay in live Jurkat cells with test compound 10. Dose-response curve for SPR (n = 2).

Having established experimental conditions that provide sensitive kinase detection with modest impact on the interaction of these kinases with reversible kinase inhibitors of interest, we utilized 3 as a probe for a quantitative target engagement assay of unrelated inhibitors in live Jurkat T-cells. Cells were treated with a compound of interest using nine different concentrations plus vehicle control, followed by incubation with 1 μM 3 for 30 min. Covalently probe-modified proteins were enriched on NeutrAvidin beads modified with biotin-tetrazine. Captured proteins were on-bead trypsinized, labeled with TMT reagents, and analyzed by quantitative mass spectrometry (Fig. 3*A*). This approach enables the determination of IC_50_s for the tested inhibitor against the set of the previously determined 126 specifically captured and competed proteins. Targets of tested compounds which are not captured by 3 are not accessible in this assay.

**Figure S3:**
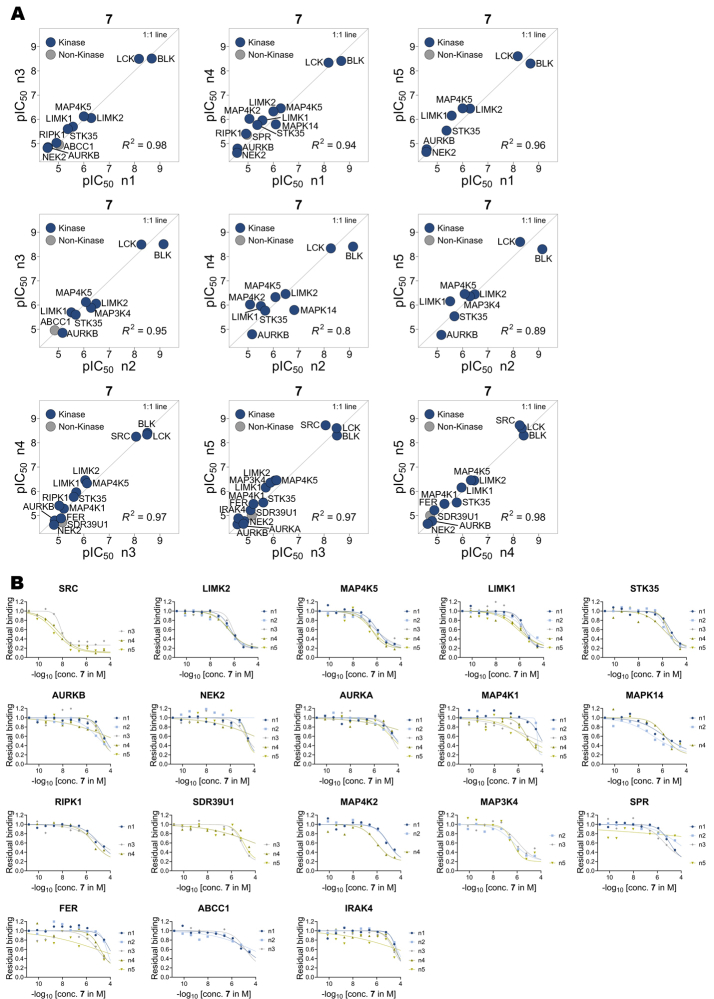


First, we confirmed the applicability of this assay using the BCR-ABL and tyrosine kinase inhibitor dasatinib (7) and the cyclin-dependent kinase inhibitor dinaciclib (8) (Fig. 3*B*). Replicates showed a very good correlation of 0.95 (7) and 0.7 (8) (Fig. 3, *C* and *E* and supplemental Table S5) demonstrating the reproducibility and robustness of the assay. To further validate robustness, the assay was repeated in additional fully independent triplicates with 7, resulting in a total of five replicates. Replicates showed a very good correlation with R^2^ values ranging from 0.8 to 0.98 for pairwise comparison across all five replicate experiments (supplemental Fig. S3*A* and supplemental Table S5), as well as the comparison of average pIC_50_s of triplicates *versus* duplicates performed a year earlier (Fig. 3*D*). For measurements passing the quality criteria, the average standard error for pIC_50_ values improved from 0.1 (n = 2) to 0.08 (n = 3) and we determined an average SD of ±0.19 over all five replicates. Exemplary dose-response curves for some of the known targets are shown in Figure 3, *F* and *G* (see also supplemental Figs. S3*B* and S4*B*). Some expected targets like the Ephrin receptors or BCR-ABL for 7 (17) were not covered, either due to a lack of expression in Jurkat cells or not being captured by 3.

When comparing the rank order of pIC_50_ values determined in the cellular target engagement assay in Jurkat cells with apparent dissociation constants (pK_d_^app^) determined with the kinobeads assay using Jurkat cellular extracts, we found excellent agreement and good correlation for 7 (Fig. 3*H* and supplemental Table S6). These values are also in good agreement to a pEC_50_, previously determined by dose-dependent reduction of the phosphorylation of target proteins in a large scale phosphoproteomics study by Zecha *et al* (Fig. 3*J*) (32). Interestingly, a systematic shift between the cellular target engagement assay and the kinobeads assay was observed for dinaciclib (Fig. 3*I*); kinobeads data showed higher affinities of 8 for target proteins.

In addition to kinases, several nonkinase proteins were also identified and specifically competed in the single concentration competition experiments (Fig. 2*C*), including SPR and ABCC1. These two proteins have not been reported in published kinobeads work and were not robustly captured in the kinobeads experiments performed in this study (see supplemental Tables S1 and S6). To validate the specific interaction of 3 with SPR and ABCC1, we performed cross-competition experiments in Jurkat cells using previously reported inhibitors (Fig. 3*B*). The EGFR kinase inhibitor EKI-785 (9) has been described to inhibit ABCC1 (33) and consistent with specific binding of ABCC1 to 3, dose-dependent competition was observed (Fig. 3*K*). Similarly, capturing of SPR by 3 was inhibited in a dose-dependent manner by the SPR inhibitor QM385 (10) (Fig. 3*L*) (34).

## Discussion

Multiple kinase profiling platforms are available to date, most of which relying on recombinantly expressed proteins that are probed in isolation. Chemoproteomics approaches enable accessing endogenously expressed kinases, and probes have been described that in principle enable profiling of kinase inhibitors in live cells; however, a robust workflow using cell-permeable kinase probes that cover a substantial range of the human kinome has been missing so far. Here, we presented and benchmarked a chemoproteomics workflow for kinase inhibitor profiling in live cells using a TCO-modified analog of the promiscuous kinase inhibitor XO44. Characterization of probes with kinobeads showed that replacing the alkyne handle with a trans-cyclooctene moiety substantially impaired kinase binding in cell extracts (Fig. 1, *C* and *D*). The difference in the binding strength of 3 *versus* 2 was less pronounced in live cells (Fig. 1*F*). This may be attributed to the higher reactivity of these compounds at 37 °C than lysate-based experiments that were performed at 4 °C.

The more efficient IEDDA reaction for enrichment (30) that requires fewer manual handling steps contributed to the improved kinase enrichment. When optimizing probe concentrations, we observed impaired binding of kinases at the highest concentrations with increasing background signal (Fig. 2*F*), suggesting that higher levels of unspecific proteome labeling at > 1 μM 3 eventually leads to saturation of the NeutrAvidin beads and thus reduction of the total kinase signal (35). Consistent with this, similar effects were observed when the experiment was performed in Jurkat cellular extract (supplemental Fig. S2, *C* and *E*). A notable exception however was that the intensity drop for kinases and nonkinase proteins at the highest concentration of 3 was only observed in experiments in Jurkat cells (Fig. 2*F*), which may be explained by inactivation of an active transporter at very high probe concentrations.

As unspecific binding of kinases might contribute to overall kinase coverage but resulting in potentially false negative readouts in competition binding experiments, we took special attention on balancing kinase coverage and signal to noise. Further we aimed to minimize the influence of the probe on the interaction of kinase inhibitors of interest with endogenously expressed kinases by keeping probe concentrations and incubation time relatively low and in the linear binding range. Further we conservatively report only those kinases as specific binders that can be competed for probe binding by excess concentrations of free inhibitor.

Comparing the rank order of pIC_50_ values determined in Jurkat cells using the cellular target engagement assay developed with the above-described considerations with apparent dissociation constants (pK_d_^app^) determined with the kinobeads assay using Jurkat cellular extracts showed a good correlation for 7, also agreeing with pEC_50_s, previously determined by dose-dependent reduction of phosphorylation of target proteins in a large scale phosphoproteomics study by Zecha *et al* (Fig. 3*J*) (32). A systematic shift between the cellular target engagement assay and the kinobeads assay was observed for dinaciclib (Fig. 3*I*); kinobeads data showed higher affinities of 8 for target proteins. As intracellular ATP levels are higher than ADP/ATP levels in cell extracts, competition with the endogenous cosubstrate may contribute to the observed shift. However, previous studies have shown that the binding strengths observed for dinaciclib for cyclin-dependent kinases varied dependent on the co-expressed cyclin (36). Cell and cell extract–based experiments do not resolve complex-specific differences in target engagement and rather provide an affinity estimate that averages across all present conformations, activation states, and complexes making interpretation of assay-dependent differences more complex. In addition, cell penetration and intracellular distribution of inhibitors can add to differences between these experiment types.

In addition to kinases, several nonkinase proteins were also identified and specifically competed by kinase inhibitors (Fig. 2*C*). The 31 nonkinase proteins captured by 3 included eight proteins described to bind to either ATP, GTP, or NAD/P, explaining their binding to the ATP-mimetic XO44. Among those proteins, the translocase multidrug resistance protein 1 (ABCC1) belonging to the ABC transporter superfamily and the oxidoreductase SPR which utilizes NADP^+^ (Fig. 2*E*) are two examples with demonstrated relevance for drug discovery as drug transporter (ABCC1) or direct drug target (SPR). Further, specific capturing of 10 hydrolases (esterases/proteases) was observed. Hydrolases contain a nucleophilic serine residue in their catalytic site and previous reports have demonstrated that sulfonyl fluorides can react with nucleophilic serines (37). The higher number of nonkinase proteins competed in the cellular assay can be attributed to the higher reactivity at 37 °C or could be explained by their location in membranes and association to lipid metabolism, as the binding site of the proteins might only be amenable to compound binding in their native environment.

We performed cross-competition experiments in Jurkat cells to validate the specific interaction of 3 with SPR and ABCC1. SPR plays an important role in the biosynthesis of tetrahydrobiopterin (BH_4_) (38) and has been suggested as potential drug target for analgesics (39). However, there are toxicity concerns as mutations in the SPR gene lead to low BH_4_ levels and have been associated with neurological deficits, such as mental retardation, abnormal movements, or hypersalivation (40). Binding of both proteins to 3 can be rationalized from crystal structures (19, 41, 42) (supplemental Fig. S4, *C* and *D*). The fact that these two off-targets bind to the promiscuous kinase probe 3 suggests that ABCC1 and SPR may be more frequently occurring kinase inhibitor off-targets with relevance for pharmacodynamics and drug safety and should therefore be included into standard off-target profiling assay cascades.

In conclusion, we presented and benchmarked a robust chemoproteomics workflow for kinase inhibitor profiling in live cells using a TCO-modified analog of the promiscuous kinase inhibitor XO44. When comparing rank-orders of kinases bound by the reference inhibitors dasatinib and dinaciclib, we found generally good agreement with the lysate-based kinobeads assay with multiple potential factors contributing to differences in IC_50_s including cosubstrate concentrations, kinase–protein complexes, as well as cell penetration and intracellular distribution of inhibitors. In addition, we describe new off-targets for kinase scaffolds such as SPR that may contribute to the activity and safety profile of drug candidates. To further enhance translational relevance of kinase inhibitor profiling, future work will be directed to adopting this workflow for patient-derived samples such as peripheral blood mononuclear cells from blood draws and *ex vivo* biopsy cultures.

## Data Availability

The mass spectrometry proteomics data have been deposited to the ProteomeXchange Consortium *via* the PRIDE (29) partner repository with the dataset identifier PXD047949.

## Supplemental data

This article contains supplemental data (11, 12, 16, 17, 19, 43).

## Conflicts of interest

U. G. and H. C. E. are employees of GSK; M. B. and H. C. E. are shareholders of GSK. The authors declare that they have no conflicts of interest with the contents of this article.

## Figures and Tables

**Figure S4:**