# Supplementary material for: A Probe-Based Target Engagement Assay for Kinases in Live Cells
Source: Mol Cell Proteomics. 2025 Apr 3;24(5):100963. doi: 10.1016/j.mcpro.2025.100963 (PMC12076712; doi:10.1016/j.mcpro.2025.100963)
Supplement: Supporting Information [file mmc7.docx]

**A probe-based target engagement assay for kinases in live cells**

Ursula M. Glocker^1^, Florian Braun^2^, H. Christian Eberl^1^* and Marcus Bantscheff^1,3^*

^1^Cellzome, a GSK company, 69117 Heidelberg, Germany.

^2^Chemical Synthesis Core Facility, European Molecular Biology Laboratory, 69117 Heidelberg, Germany.

^3^Present address: pRED, F. Hoffmann-La Roche AG, 4070 Basel, Switzerland

*Corresponding Authors

hans-christian.h.eberl@gsk.com; marcus.bantscheff@roche.com

**Table of contents**

Supplemental methods: Chemical synthesis and characterization.

Supplemental methods: Chemical synthesis schemes.

Supplemental note: Considerations to optimize probe concentration and labeling time.

Figure S1. Characterization of probes with kinobeads.

Figure S2. Characterization of compound **3**.

Figure S3 Kinase target engagement with Dasatinib in n=5 using compound **3**.

Figure S4. Kinome coverage and rationale for capturing of non-kinases.

Table S1. Characterization of probes with kinobeads.

Table S2. Jurkat expression proteome.

Table S3. Single concentration competition.

Table S4. Assay optimization.

Table S5. Target engagement assays using **3**.

Table S6. Kinobeads of kinase inhibitors Dasatinib and Dinaciclib.

**Supporting Information**

**Methods**

**Chemical synthesis and characterization**

**General methods**

Reagents were purchased from Sigma Aldrich (Germany), Enamine (Ukraine) and abcr (Germany) and used without further purification. All solvents, including anhydrous solvents, were used as obtained from the commercial sources. Air and water-sensitive reagents and reactions were generally handled under argon atmosphere. The reaction progress was monitored by TLC on Merck silica gel plates 60 F254. Detection was executed with a UV-Kabinett HP-UVIS (biostep) at 254 nm or with potassium permanganate staining. Flash chromatographic purification was performed on a Biotage® Isolera One purification system using Biotage® SFär C18 D flash cartridges. Nuclear magnetic resonance spectra were recorded on a Bruker Avance (400 MHz) NMR System at 298 K. Chemical shifts (δ) are given in parts per million (ppm), coupling constants (J) given in Hertz (Hz) and multiplicity is reported using standard abbreviations. Only signals for the main conformers are reported. UHPLC/MS analyses were performed on Agilent 1290 series equipment consisting of an Agilent 1290 quaternary pump, a 1290 sampler, a 1290 thermostated column compartment and a 1290 Diode array detector VL+ equipped with a quadrupole LC/MS 6120 and an Infinity 1260 ELSD. The analytical column used was a Titan C18 UHPLC Column (2.1 X 30 mm, 1.9 µm) operated at 40 °C and 1.5 ml/min flow rate with a gradient (10% to 15% B in 0.4 min, 15% to 100% B in 1.6 min, 100% B for 0.5 min) using water (A) and acetonitrile (B), both containing 0.1% TFA as solvents. Compound purity was determined by ELSD monitoring. 2-Chloro-6-((5-cyclopropyl-1H-pyrazol-3-yl)amino)pyrimidine-4-carboxylic acid **(1c)** was synthesized following the literature known procedure^1^.

**2-Chloro-6-((5-cyclopropyl-1*H*-pyrazol-3-yl)amino)-*N*-ethylpyrimidine-4-carboxamide (1b)**

To a mixture of **1c** (559 mg, 2.00 mmol) and ethanamine (1.0 mL, 2.00 mmol) in DMF (8.0 mL) was added 1-[bis(dimethylamin)methylen]-1*H*-1,2,3-triazol[4,5-b]pyridinium-3-oxid-hexafluorophosphat (989 mg, 2.60 mmol) followed by DIPEA (1.40 mL, 8.00 mmol) and the reaction mixture was stirred at room temperature overnight. The mixture was concentrated under reduced pressure and the crude material was purified on a Biotage® SFär C18 D – 30 g column using a water/methanol gradient to obtain 512 mg (83%) of the title compound.

**^1^H NMR (400 MHz, DMSO-*d*_6_):** δ 12.30 (bs, 0.5H), 12.25 (bs, 0.5H), 10.74 (bs, 0.5H), 10.53 (bs, 0.5H), 8.66 (t, *J* = 6.1 Hz, 1H), 8.19 (bs, 0.5H), 7.31 (bs, 0.5H), 6.39 (bs, 0.5H), 5.67 (bs, 0.5H), 3.32 – 3.23 (m, 2H), 1.97 – 1.83 (m, 1H), 1.09 (t, *J* = 7.2 Hz, 3H), 0.97 – 0.88 (m, 2H), 0.73 – 0.64 (m, 2H).

**^13^C NMR (101 MHz, DMSO-*d*_6_):** δ 162.0, 34.3, 15.1, 7.2.

**UHPLC**: t*_R_* = 1.33 min, **MS (ESI):** 307 (M + H)^+^.

***Tert*-butyl 4-(4-((5-cyclopropyl-1*H*-pyrazol-3-yl)amino)-6-(ethylcarbamoyl)pyrimidin-2-yl)piperazine-1-carboxylate (1a)**

A mixture of compound **(1b)** (153 mg, 0.50 mmol) and *tert*-butyl piperazine-1-carboxylate (186 mg, 1.00 mmol) in DMF (8.0 mL) was reacted at 100 °C overnight. The mixture was concentrated under reduced pressure and the crude material was purified on a Biotage® SFär C18 D – 12 g column using a water/methanol gradient to obtain 181 mg (79%) of the title compound.

**^1^H NMR (400 MHz, DMSO-*d*_6_):** δ 9.79 (bs, 1H), 8.58 (t, *J* = 6.2 Hz, 1H), 6.79 (bs, 1H), 6.19 (bs, 1H), 3.76 (bs, 4H), 3.41 (bs, 4H), 3.31 – 3.22 (m, 2H), 1.95 – 1.83 (m, 1H), 1.43 (s, 9H), 1.10 (t, *J* = 7.1 Hz, 3H), 0.98 – 0.87 (m, 2H), 0.73 – 0.62 (m, 2H).

**^13^C NMR (101 MHz, DMSO-*d*_6_):** δ 163.4, 160.7, 154.0, 147.9, 145.5, 92.9, 79.0, 43.4, 33.5, 28.1, 14.9, 7.8, 6.8.

**UHPLC**: t*_R_* = 1.60 min, **MS (ESI):** 457 (M + H)^+^.

**4-((4-(4-((5-Cyclopropyl-1*H*-pyrazol-3-yl)amino)-6-(ethylcarbamoyl)pyrimidin-2-yl)piperazin-1-yl)methyl)benzenesulfonyl fluoride (1)**

Compound **(1a)** (91 mg, 0.20 mmol) was treated with 4 M HCl in Dioxane (1.0 mL, 4.00 mmol) at room temperature for 1 h. All volatiles were removed under reduced pressure and the residue was dissolved in DMF (2.0 mL). 4-(Bromomethyl)benzenesulfonyl fluoride (56 mg, 0.22 mmol) was added followed by DIPEA (140 µL, 0.80 mmol) and the reaction mixture was stirred at room temperature for 30 min. The mixture was concentrated under reduced pressure and the crude material was purified on a Biotage® SFär C18 D – 12 g column using a water/ methanol gradient to obtain 62 mg (59%) of the analytically pure title compound.

**^1^H NMR (400 MHz, DMSO-*d*_6_):** δ 12.01 (bs, 1H), 9.75 (bs, 1H), 8.54 (t, *J* = 6.2 Hz, 1H), 8.12 (d, *J* = 8.4 Hz, 2H), 7.78 (d, *J* = 8.2 Hz, 2H), 6.77 (bs, 1H), 6.10 (bs, 1H), 3.81 (bs, 4H), 3.71 (s, 2H), 3.31 – 3.22 (m, 2H), 2.49 – 2.44 (m, 4H), 1.91 – 1.82 (m, 1H), 1.08 (t, *J* = 7.1 Hz, 3H), 0.94 – 0.85 (m, 2H), 0.68 – 0.60 (m, 2H).

**^13^C NMR (101 MHz, DMSO-*d*_6_):** δ 163.5, 160.8, 148.3, 130.3, 129.9, 129.8, 128.5,92.8, 61.1, 52.6, 43.6, 33.5, 14.9, 7.8.

**UHPLC**: t*_R_* = 1.33 min, **MS (ESI):** 529 (M + H)^+^.

***Tert*-butyl (2-(2-chloro-6-((5-cyclopropyl-1*H*-pyrazol-3-yl)amino)pyrimidine-4-carboxamido)ethyl)carbamate (3d)**

To a mixture of **1c** (622 mg, 2.00 mmol) and *tert*-butyl (2-aminoethyl)carbamate (320 mg, 2.00 mmol) in DMF (8.0 mL) was added 1-[bis(dimethylamin)methylen]-1*H*-1,2,3-triazol[4,5-b]pyridinium-3-oxid-hexafluorophosphat (989 mg, 2.60 mmol) followed by DIPEA (1.40 mL, 8.00 mmol) and the reaction mixture was stirred at room temperature overnight. The mixture was concentrated under reduced pressure and the crude material was purified on a Biotage® SFär C18 D – 30 g column using a water/methanol gradient to obtain 604 mg (72%) of the title compound.

**^1^H NMR (400 MHz, DMSO-*d*_6_):** δ 12.32 (bs, 0.5 H), 12.24 (bs, 0.5 H), 10.76 (bs, 0.5 H), 10.54 (bs, 0.5 H), 8.65 (t, *J* = 5.6 Hz, 1H), 8.20 (bs, 0.5 H), 7.32 (bs, 0.5 H), 6.92 (t, *J* = 5.4 Hz, 1H), 6.36 (bs, 0.5 H), 5.66 (bs, 0.5 H), 3.34 – 3.26 (m, 2H), 3.13 – 3.04 (m, 2H), 1.95 – 1.84 (m, 1H), 1.36 (s, 9H), 0.97 – 0.89 (m, 2H), 0.72 – 0.65 (m, 2H).

**^13^C NMR (101 MHz, DMSO-*d*_6_):** δ 162.1, 155.8, 77.7, 39.4, 39.3, 28.2, 7.8, 6.7.

**UHPLC**: t*_R_* = 1.53 min, **MS (ESI):** 422 (M + H)^+^.

**Benzyl 4-(4-((2-((tert-butoxycarbonyl)amino)ethyl)carbamoyl)-6-((5-cyclopropyl-1*H*-pyrazol-3-yl)amino)pyrimidin-2-yl)piperazine-1-carboxylate (3c)**

A mixture of compound **(3d)** (386 mg, 0.92 mmol) and benzyl piperazine-1-carboxylate (403 mg, 1.83 mmol) in DMF (15 mL) was reacted at 100 °C overnight. The mixture was concentrated under reduced pressure and the crude material was purified on a Biotage® SFär C18 D – 30 g column using a water/methanol gradient to obtain 485 mg (88%) of the title compound.

**^1^H NMR (400 MHz, DMSO-*d*_6_):** δ 12.03 (s, 1H), 9.82 (bs, 1H), 8.69 (t, *J* = 5.6 Hz, 1H), 7.42 – 7.37 (m, 4H), 7.37 – 7.29 (m, 2H), 7.00 (t, *J* = 5.5 Hz, 1H), 6.78 (bs, 1H), 6.21 (bs, 1H), 5.12 (s, 2H), 3.82 (bs, 3H), 3.51 (bs, 4H), 3.32 – 3.24 (m, 2H), 3.14 – 3.05 (m, 4H), 1.94 – 1.85 (m, 1H), 1.36 (s, 9H), 0.96 – 0.88 (m, 2H), 0.71 – 0.65 (m, 2H).

**^13^C NMR (101 MHz, DMSO-*d*_6_):** δ 163.8, 160.7, 156.1 154.6, 136.9, 128.5, 127.9, 127.6, 77.8, 66.4, 43.3, 39.4, 39.3, 28.2, 7.9, 6.8.

**UHPLC**: t*_R_* = 1.69 min, **MS (ESI):** 606 (M + H)^+^.

***Tert-*butyl (2-(6-((5-cyclopropyl-1H-pyrazol-3-yl)amino)-2-(piperazin-1-yl)pyrimidine-4-carboxamido)ethyl)carbamate (3b)**

To a solution of compound **(3c)** (245 mg, 0.40 mmol) in methanol (40 mL) was added 10% Pd/C (19 mg, 20 mol%) and the reaction mixture was stirred at 30 °C under 1 atm. of hydrogen for 5 h. After completion of the reaction the mixture was filtered through a small plug of celite and the solvent was removed under reduced pressure. The title compound was used in next step without further purification.

**^1^H NMR (400 MHz, DMSO-*d*_6_):** δ 12.07 (bs, 1H), 9.75 (bs, 1H), 8.63 (t, *J* = 6.1 Hz, 1H), 6.99 (t, *J* = 5.7 Hz, 1H), 6.77 (bs, 1H), 6.15 (bs, 1H), 3.77 (bs, 1H), 3.71 (bs, 4H), 3.31 – 3.22 (m, 2H), 3.14 – 3.02 (m, 2H), 2.75 (bs, 3H), 2.36 (m, 1H), 1.93 – 1.81 (m, 1H), 1.37 (s, 9H), 0.96 – 0.87 (m, 1H), 0.69 – 0.61 (m, 1H).

**^13^C NMR (101 MHz, DMSO-*d*_6_):** δ 164.0, 160.9, 156p.1, 92.6, 77.8, 54.6, 45.9, 45.6, 44.7, 43.5, 39.3, 28.2, 7.83, 6.92.

**UHPLC**: t*_R_* = 1.14 min, **MS (ESI):** 472 (M + H)^+^.

***Tert*-butyl (2-(6-((5-cyclopropyl-1*H*-pyrazol-3-yl)amino)-2-(4-(4-(fluorosulfonyl)benzyl)piperazin-1-yl)pyrimidine-4-carboxamido)ethyl)carbamate (3a)**

To a solution of compound **(3b)** (91 mg, 0.19 mmol) and 4-(bromomethyl)benzenesulfonyl fluoride (54 mg, 0.21 mmol) in DMF (1.9 mL) was added DIPEA (101 µL, 0.58 mmol) and the reaction mixture was stirred at room temperature for 30 min. The mixture was concentrated under reduced pressure and the crude material was purified on a Biotage® SFär C18 D – 12 g column using a water/ methanol gradient to obtain 80 mg (64%) of the title compound.

**^1^H NMR (400 MHz, DMSO-*d*_6_):** δ 12.00 (bs, 1H), 9.78 (bs, 1H), 8.65 (t, *J* = 5.9 Hz, 1H), 8.12 (d, *J* = 8.5 Hz, 2H), 7.78 (d, *J* = 8.5 Hz, 2H), 6.99 (t, *J* = 5.7 Hz, 1H), 6.76 (bs, 1H), 6.16 (bs, 1H), 3.82 (bs, 4H), 3.71 (s, 2H), 3.31 – 3.21 (m, 2H), 3.13 – 3.03 (m, 2H), 2.46 (bs, 4H), 1.92 – 1.80 (m, 1H), 1.34 (s, 9H), 0.94 – 0.85 (m, 1H), 0.68 – 0.60 (m, 1H).

**^13^C NMR (101 MHz, DMSO-*d*_6_):** δ 163.9, 160.7, 156.1, 148.3, 130.4, 130.0, 129.8, 128.5, 77.8, 61.1, 52.6, 43.6, 39.6, 39.4, 28.2, 7.8, 6.5.

**UHPLC**: t*_R_* = 1.47 min, **MS (ESI):** 644 (M + H)^+^.

***(E*)-Cyclooct-4-en-1-yl-(2-(6-((5-cyclopropyl-1*H*-pyrazol-3-yl)amino)-2-(4-(4-(fluorosulfonyl)benzyl)-piperazin-1-yl)pyrimidine-4-carboxamido)ethyl)carbamate (3)**

Compound **3a** (66 mg, 0.10 mmol) was treated with 4 M HCl in Dioxane (0.51 mL, 2.05 mmol) at room temperature for 1 h. All volatiles were removed under reduced pressure and the residue was dissolved in DMF (1.0 mL). TCO4 / A- active ester (SC-8019; SiChem) (30 mg, 0.10 mmol) was added followed by DIPEA (72 µL, 0.41 mmol) and the reaction mixture was stirred at room temperature for 3 h. The mixture was concentrated under reduced pressure and the crude material was purified on a Biotage® SFär C18 D – 12 g column using a water/ methanol gradient to obtain 32 mg (45%) of the analytically pure title compound.

**^1^H NMR (400 MHz, DMSO-*d*_6_):** δ 12.01 (s, 1H), 9.78 (bs, 1H), 8.62 (t, *J* = 5.6 Hz, 1H), 8.12 (d, *J* = 8.1 Hz, 2H), 7.77 (d, *J* = 8.1 Hz, 2H), 7.30 (t, *J* = 5.7 Hz, 1H), 6.77 (bs, 1H), 6.21 (bs, 1H), 5.64 – 5.53 (m, 1H), 5.51 – 5.39 (m, 1H), 4.72 – 4.62 (m, 1H), 3.81 (bs, 4H), 3.70 (s, 2H), 3.34 – 3.25 (m, 2H), 3.20 – 3.11 (m, 2H), 2.46 (bs, 4H), 2.27 – 2.14 (m, 1H), 2.14 – 2.00 (m, 3H), 2.00-1.92 (m, 1H), 1.92 – 1.82 (m, 1H), 1.80 – 1.66 (m, 1H), 1.63 – 1.48 (m, 2H), 1.47 – 1.33 (m, 1H), 1.15 – 1.04 (m, 1H), 0.93 – 0.85 (m, 2H), 0.68 – 0.60 (m, 2H).

**^13^C NMR (101 MHz, DMSO-*d*_6_):** δ 163.9, 160.7, 156.3, 148.3, 135.0, 131.4, 130.4, 129.9, 129.8, 128.5, 95.9, 68.5, 61.1, 52.6, 43.6, 40.4, 39.5, 39.3, 33.9, 32.1, 29.6, 27.4, 7.8, 6.5.

**UHPLC**: t*_R_* = 1.52 min, **MS (ESI):** 696 (M + H)^+^.

**Methyl 2-chloro-6-((5-methyl-1H-pyrazol-3-yl)amino)pyrimidine-4-carboxylate (4d)**

To a solution of methyl 2,6-dichloropyrimidine-4-carboxylate (5.18 g, 25 mmol) in THF (50 mL) at 0 °C, was added a mixture of 5-methyl-1*H*-pyrazol-3-amine (2.43 g, 25 mmol) and *N*,*N*-diisopropylethylamine (5.2 mL, 30 mmol) in THF (50 mL) and the reaction was stirred at room temperature overnight. The solvent was removed under reduced pressure and the residue was heated in methanol (80 mL) at 80 °C for 1 h. The mixture was cooled to room temperature and the precipitate was filtered and dried under vacuum at 40 °C overnight to obtain 6.69 g (77%) of the title compound.

**^1^H NMR (400 MHz, DMSO-*d*_6_):** δ 12.29 (bs, 1H), 12.23 (bs, 1H), 10.82 (bs, 0.5H), 10.69 (bs, 0.5H), 8.29 (bs, 0.5H), 7.34 (bs, 0.5H), 6.47 (bs, 0.5H), 5.77 (bs, 0.5H), 2.23 (s, 3H), **UHPLC**: t*_R_* = 0.66 min, **MS (ESI):** 268 (M + H)^+^.

**2-Chloro-6-((5-methyl-1H-pyrazol-3-yl)amino)pyrimidine-4-carboxylic acid (4c)**

To a solution of compound **4d** (2.68 g, 10.0 mmol) in Dioxane (20 mL), 1 M NaOH (20 mL) was slowly added and the reaction mixture was stirred at room temperature for 30 min. The mixture was acidified to pH 4 with 1 M HCl and the white precipitate was filtered of, washed with water and dried under vacuum at 40 °C overnight to obtain 2.54 g (100 %) of the analytically pure title compound.

**^1^H NMR (400 MHz, DMSO-*d*_6_):** δ 12.72 (bs, 1H), 10.77 (bs, 0.5H), 10.60 (bs, 0.5H), 8.25 (bs, 0.5H), 7.33 (bs, 0.5H), 6.46 (bs, 0.5H), 5.77 (bs, 0.5H), 2.23 (s, 3H), **UHPLC**: t*_R_* = 0.31 min, **MS (ESI):** 254 (M + H)^+^.

**2-Chloro-*N*-ethyl-6-((5-methyl-1*H*-pyrazol-3-yl)amino)pyrimidine-4-carboxamide** **(4b)**

To a mixture of **4c** (507 mg, 2.00 mmol) and ethanamine (1.0 mL, 2.00 mmol) in DMF (8.0 mL) was added 1-[bis(dimethylamin)methylen]-1*H*-1,2,3-triazol[4,5-b]pyridinium-3-oxid-hexafluorophosphat (989 mg, 2.60 mmol) followed by DIPEA (1.40 mL, 8.00 mmol) and the reaction mixture was stirred at room temperature overnight. The mixture was concentrated under reduced pressure and the crude material was purified on a Biotage® SFär C18 D – 30 g column using a water/methanol gradient to obtain 439 mg (78%) of the title compound.

**^1^H NMR (400 MHz, DMSO-*d*_6_):** δ 12.25 (bs, 0.5H), 12.21 (bs, 0.5H), 10.75 (bs, 0.5H), 10.53 (bs, 0.5H), 8.66 (t, *J* = 6.2 Hz, 1H), 8.18 (bs, 0.5H), 7.32 (bs, 0.5H), 6.47 (bs, 0.5H), 5.78 (bs, 0.5H), 3.33 – 3.22 (m, 2H), 2.23 (s, 3H), 1.09 (t, *J* = 7.2 Hz, 3H).

**^13^C NMR (101 MHz, DMSO-*d*_6_):** δ 162.0, 34.3, 15.1, 11.1.

**UHPLC**: t*_R_* = 0.99 min, **MS (ESI):** 281 (M + H)^+^.

***Tert*-butyl 4-(4-(ethylcarbamoyl)-6-((5-methyl-1*H*-pyrazol-3-yl)amino)pyrimidin-2-yl)piperazine-1-carboxylate (4a)**

A mixture of compound **4b** (140 mg, 0.50 mmol) and *tert*-butyl piperazine-1-carboxylate (186 mg, 1.00 mmol) in DMF (8.0 mL) was reacted at 100 °C overnight. The mixture was concentrated under reduced pressure and the crude material was purified on a Biotage® SFär C18 D – 12 g column using a water/methanol gradient to obtain 159 mg (74%) of the title compound.

**^1^H NMR (400 MHz, DMSO-*d*_6_):** δ 11.98 (bs, 1H), 9.78 (bs, 1H), 8.58 (t, *J* = 6.2 Hz, 1H), 6.80 (bs, 1H), 6.27 (bs, 1H), 3.77 (bs, *J* = 5.4 Hz, 4H), 3.42 (bs, 4H), 3.31 – 3.22 (m, 2H), 2.22 (s, 3H), 1.43 (s, 9H), 1.10 (t, *J* = 7.1 Hz, 3H).

**^13^C NMR (101 MHz, DMSO-*d*_6_):** δ 163.4, 160.8, 154.0, 148.1, 138.4, 95.6, 79.0, 43.4, 33.5, 28.1, 14.9, 10.8.

**UHPLC**: t*_R_* = 1.53 min, **MS (ESI):** 431 (M + H)^+^.

**4-((4-(4-(Ethylcarbamoyl)-6-((5-methyl-1*H*-pyrazol-3-yl)amino)pyrimidin-2-yl)piperazin-1-yl)methyl)benzenesulfonyl fluoride (4)**

Compound **4a** (86 mg, 0.20 mmol) was treated with 4 M HCl in Dioxane (1.0 mL, 4.00 mmol) at room temperature for 1 h. All volatiles were removed under reduced pressure and the residue was dissolved in DMF (2.0 mL). 4-(Bromomethyl)benzenesulfonyl fluoride (56 mg, 0.22 mmol) was added followed by DIPEA (140 µL, 0.80 mmol) and the reaction mixture was stirred at room temperature for 30 min. The mixture was concentrated under reduced pressure and the crude material was purified on a Biotage® SFär C18 D – 12 g column using a water/ methanol gradient to obtain 36 mg (36%) of the analytically pure title compound.

**^1^H NMR (400 MHz, DMSO-*d*_6_):** δ 11.96 (bs, 1H), 9.75 (bs, 1H), 8.54 (t, *J* = 6.2 Hz, 1H), 8.12 (d, *J* = 8.5 Hz, 2H), 7.78 (d, *J* = 8.2 Hz, 2H), 6.77 (bs, 1H), 6.21 (bs, 1H), 3.81 (bs, 4H), 3.71 (s, 2H), 3.31 – 3.21 (m, 2H), 2.50 – 2.43 (m, 4H), 2.19 (s, 3H), 1.09 (t, *J* = 7.1 Hz, 3H).

**^13^C NMR (101 MHz, DMSO-*d*_6_):** δ 163.5, 160.8, 148.3, 130.4, 130.0, 129.8, 128.5, 95.5, 61.1, 52.6, 43.6, 33.5, 14.9.

**UHPLC**: t*_R_* = 1.90 min, **MS (ESI):** 503 (M + H)^+^.

**2-Chloro-6-((5-methyl-1H-pyrazol-3-yl)amino)-*N*-(prop-2-yn-1-yl)pyrimidine-4-carboxamide (5b)**

To a mixture of **(4c)** (880 mg, 3.47 mmol) and prop-2-yn-1-amine (191 mg, 3.47 mmol) in DMF (14 mL) was added 1-[bis(dimethylamin)methylen]-1*H*-1,2,3-triazol[4,5-b]pyridinium-3-oxid-hexafluorophosphat (1.72 g, 4.51 mmol) followed by DIPEA (2.42 mL, 13.9 mmol) and the reaction mixture was stirred at room temperature overnight. The mixture was concentrated under reduced pressure and water was added to the residue. The crude product was extracted with ethyl acetate containing a minimum amount of methanol. The combined organics were washed with brine, dried of magnesium sulfate, filtered and evaporated under reduced pressure. The crude material was purified on a Biotage® SFär C18 D – 30 g column using a water/methanol gradient to obtain 757 mg (75%) of the title compound.

**^1^H NMR (400 MHz, DMSO-*d*_6_):** δ 12.28 (bs, 0.5H), 12.21 (bs, 0.5H), 10.79 (bs, 0.5H), 10.59 (bs, 0.5H), 9.05 (d, *J* = 6.0 Hz, 1H), 8.21 (bs, 1H), 7.33 (bs, 1H), 6.47 (bs, 1H), 5.78 (bs, 1H), 4.01 (dd, *J* = 6.0, 2.5 Hz, 2H), 3.10 (d, *J* = 2.5 Hz, 1H), 2.23 (s, 3H),

**UHPLC**: t*_R_* = 1.02 min, **MS (ESI):** 291 (M + H)^+^.

***Tert*-butyl 4-(4-((5-methyl-1*H*-pyrazol-3-yl)amino)-6-(prop-2-yn-1-ylcarbamoyl)pyrimidin-2-yl)piperazine-1-carboxylate (5a)**

A mixture of compound **5b** (145 mg, 0.50 mmol) and *tert*-butyl piperazine-1-carboxylate (186 mg, 1.00 mmol) in DMF (8.0 mL) was reacted at 100 °C for 3 h. The mixture was concentrated under reduced pressure and the crude material was purified on a Biotage® SFär C18 D – 12 g column using a water/methanol gradient to obtain 135 mg (61%) of the title compound.

**^1^H NMR (400 MHz, DMSO-*d*_6_):** δ 11.99 (s, 1H), 9.84 (bs, 1H), 8.95 (t, *J* = 6.1 Hz, 1H), 6.78 (bs, 1H), 6.30 (bs, 1H), 4.01 (dd, *J* = 6.1, 2.5 Hz, 2H), 3.79 (bs, 4H), 3.41 (bs, 4H), 3.10 (d, *J* = 2.5 Hz, 1H), 2.22 (s, 3H), 1.43 (s, 9H), **^13^C NMR (DMSO-*d*_6_):** δ 163.6, 160.8, 154.0, 148.0, 138.4, 95.6, 81.3, 79.0, 72.6, 43.4, 28.2, 28.1, 10.8, **UHPLC**: t*_R_* = 1.54 min, **MS (ESI):** 441 (M + H)^+^.

**4-((4-(4-((5-Methyl-1H-pyrazol-3-yl)amino)-6-(prop-2-yn-1-ylcarbamoyl)pyrimidin-2-yl)piperazin-1-yl)methyl)benzenesulfonyl fluoride (5)**

A solution of compound **5a** (128 mg, 0.29 mmol) in DCM (3.0 mL) was treated with TFA (1.0 mL) at room temperature for 1 h. All volatiles were removed under reduced pressure and the residue was dissolved in DMF (3.0 mL). 4-(Bromomethyl)benzenesulfonyl fluoride (81 mg, 0.32 mmol) was added followed by DIPEA (203 µL, 1.16 mmol) and the reaction mixture was stirred at room temperature for 30 min. The mixture was concentrated under reduced pressure and the crude material was purified on a Biotage® SFär C18 D – 12 g column using a water/ methanol gradient to obtain 73 mg (49%) of the analytically pure title compound.

**^1^H NMR (400 MHz, DMSO-*d*_6_):** δ 11.98 (s, 1H), 9.81 (bs, 1H), 8.91 (t, *J* = 5.8 Hz, 1H), 8.12 (d, *J* = 8.0 Hz, 2H), 7.78 (d, *J* = 8.0 Hz, 2H), 6.77 (bs, 1H), 6.26 (bs, 1H), 4.00 (dd, *J* = 5.8, 2.0 Hz, 2H), 3.82 (bs, 4H), 3.71 (s, 2H), 3.10 (t, *J* = 2.0 Hz, 1H), 2.48 (bs, 4H), 2.19 (s, 3H).

**^13^C NMR (101 MHz, DMSO-*d*_6_):** δ 163.7, 160.8, 148.3, 148.1, 138.3, 130.4, 128.5, 95.6, 81.3, 72.6, 61.1, 52.6, 43.6, 28.2, 10.8.

**UHPLC**: t*_R_* = 1.21 min, **MS (ESI):** 513 (M + H)^+^.

***Tert*-butyl (2-(2-chloro-6-((5-methyl-1*H*-pyrazol-3-yl)amino)pyrimidine-4-carboxamido)ethyl)carbamate (6d)**

To a mixture of **4c** (507 mg, 2.00 mmol) and *tert*-butyl (2-aminoethyl)carbamate (320 mg, 2.00 mmol) in DMF (8.0 mL) was added 1-[bis(dimethylamin)methylen]-1*H*-1,2,3-triazol[4,5-b]pyridinium-3-oxid-hexafluorophosphat (989 mg, 2.60 mmol) followed by DIPEA (1.40 mL, 8.00 mmol) and the reaction mixture was stirred at room temperature overnight. The mixture was concentrated under reduced pressure and the crude material was purified on a Biotage® SFär C18 D – 30 g column using a water/methanol gradient to obtain 505 mg (64%) of the title compound.

**^1^H NMR (400 MHz, DMSO-*d*_6_):** δ 12.21 (bs, 1H), 10.76 (bs, 0.5H), 10.55 (s, 0.5H), 8.64 (s, 1H), 8.19 (bs, 0.5H), 7.33 (bs, 0.5H), 6.92 (t, *J* = 5.6 Hz, 1H), 6.47 (bs, 0.5H), 5.77 (bs, 0.5H), 3.34 – 3.27 (m, 2H), 3.15 – 3.04 (m, 2H), 2.23 (s, 3H), 1.36 (s, 9H).

**^13^C NMR (101 MHz, DMSO-*d*_6_):** δ 162.5, 156.2, 78.2, 39.8, 39.7, 28.7, 11.1.

**UHPLC**: t*_R_* = 1.42 min, **MS (ESI):** 396 (M + H)^+^.

**Benzyl 4-(4-((2-((tert-butoxycarbonyl)amino)ethyl)carbamoyl)-6-((5-methyl-1*H*-pyrazol-3-yl)amino)pyrimidin-2-yl)piperazine-1-carboxylate (6c)**

A mixture of compound **6d** (467 mg, 1.18 mmol) and benzyl piperazine-1-carboxylate (520 mg, 2.36 mmol) in DMF (19 mL) was reacted at 100 °C for 8 h. The mixture was concentrated under reduced pressure and the crude material was purified on a Biotage® SFär C18 D – 30 g column using a water/methanol gradient to obtain 539 mg (79%) of the title compound.

**^1^H NMR (400 MHz, DMSO-*d*_6_):** δ 11.99 (bs, 1H), 9.84 (bs, 1H), 8.69 (t, *J* = 5.8 Hz, 1H), 7.52 – 7.27 (m, 6H), 7.01 (t, *J* = 5.7 Hz, 1H), 6.77 (bs, 1H), 6.31 (bs, 1H), 5.12 (s, 2H), 3.81 (bs, *J* = 5.1 Hz, 4H), 3.49 (bs, *J* = 15.2 Hz, 4H), 3.33 – 3.24 (m, 2H), 3.14 – 3.04 (m, 2H), 2.22 (s, 3H), 1.36 (s, 9H).

**^13^C NMR (101 MHz, DMSO-*d*_6_):** δ 163.9, 160.7, 156.1, 154.5, 136.8, 128.5, 127.9, 127.6, 77.8, 66.4, 43.3, 39.6, 39.4, 28.2.

**UHPLC**: t*_R_* = 1.67 min, **MS (ESI):** 580 (M + H)^+^.

***Tert*-butyl (2-(6-((5-methyl-1*H*-pyrazol-3-yl)amino)-2-(piperazin-1-yl)pyrimidine-4-carboxamido)ethyl)carbamate (6b)**

To a solution of compound **6c** (517 mg, 0.89 mmol) in methanol (9.0 mL) was added 10% Pd/C (19 mg, 20 mol%) and the reaction mixture was stirred at room temperature under 1 atm. of hydrogen overnight. After completion of the reaction the mixture was filtered through a small plug of celite and the solvent was removed under reduced pressure. The title compound was used in next step without further purification.

**^1^H NMR (400 MHz, DMSO-*d*_6_):** δ 11.98 (bs, 1H), 9.72 (bs, 1H), 8.62 (t, *J* = 5.8 Hz, 1H), 6.99 (t, *J* = 5.6 Hz, 1H), 6.79 (bs, 1H), 6.24 (bs, 1H), 3.71 (bs, *J* = 5.0 Hz, 4H), 3.33 – 3.22 (m, 2H), 3.14 – 3.03 (m, 2H), 2.73 (bs, 4H), 2.21 (s, 3H), 1.37 (s, 9H).

**^13^C NMR (101 MHz, DMSO-*d*_6_):** δ 164.0, 161.0, 156.1, 95.5, 77.8, 45.7, 44.8, 39.7, 39.5, 28.2.

**UHPLC**: t*_R_* = 0.83 min, **MS (ESI):** 446 (M + H)^+^.

***Tert*-butyl (2-(2-(4-(4-(fluorosulfonyl)benzyl)piperazin-1-yl)-6-((5-methyl-1*H*-pyrazol-3-yl)amino)pyrimidine-4-carboxamido)ethyl)carbamate trifluoroacetate (6a)**

To a solution of compound **6b** (178 mg, 0.40 mmol) and 4-(bromomethyl)benzenesulfonyl fluoride (111 mg, 0.44 mmol) in DMF (4.0 mL) was added DIPEA (210 µL, 1.20 mmol) and the reaction mixture was stirred at room temperature for 30 min. The mixture was concentrated under reduced pressure and the crude material was purified on a Biotage® SFär C18 D – 12 g column using a water/ methanol gradient to obtain 212 mg (86%) of the title compound.

**^1^H NMR (400 MHz, DMSO-*d*_6_):** δ 11.97 (bs, 1H), 9.79 (bs, 1H), 8.65 (t, *J* = 5.8 Hz, 1H), 8.12 (d, *J* = 8.2 Hz, 2H), 7.78 (d, *J* = 8.1 Hz, 2H), 6.99 (t, *J* = 5.6 Hz, 1H), 6.76 (bs, 1H), 6.20 (bs, 1H), 3.82 (bs, 4H), 3.71 (s, 2H), 3.33 – 3.23 (m, 2H), 3.14 – 3.04 (m, 2H), 2.54 – 2.39 (m, 4H), 2.19 (s, 3H), 1.34 (s, 9H).

**^13^C NMR (101 MHz, DMSO-*d*_6_):** δ 164.4, 161.2, 156.5, 148.7, 130.8, 130.5, 130.3, 128.9, 96.0, 78.2, 61.6, 53.1, 44.0, 28.6.

**UHPLC**: t*_R_* = 1.40 min, **MS (ESI):** 618 (M + H)^+^.

**(*E*)-Cyclooct-4-en-1-yl-(2-(2-(4-(4-(fluorosulfonyl)benzyl)piperazin-1-yl)-6-((5-methyl-1*H*-pyrazol-3-yl)amino)pyrimidine-4-carboxamido)ethyl)carbamate (6)**

Compound **6a** (197 mg, 0.32 mmol) was treated with 4 M HCl in Dioxane (1.6 mL, 6.40 mmol) at room temperature for 1 h. All volatiles were removed under reduced pressure and the residue was dissolved in DMF (3.0 mL). TCO4 / A- active ester (SC-8019; SiChem) (93 mg, 0.32 mmol) was added followed by DIPEA (223 µL, 1.28 mmol) and the reaction mixture was stirred at room temperature for 6 h. The mixture was concentrated under reduced pressure and the crude material was purified on a Biotage® SFär C18 D – 12 g column using a water/acetonitrile gradient (both containing 0.1% TFA) to obtain 75 mg (30%) of the analytically pure title compound.

**^1^H NMR (400 MHz, DMSO-*d*_6_):** δ 10.49 (bs, 1H), 9.99 (bs, 1H), 8.75 (t, *J* = 5.8 Hz, 1H), 8.30 (d, *J* = 8.3 Hz, 2H), 7.93 (d, *J* = 8.2 Hz, 2H), 7.33 (t, *J* = 5.8 Hz, 1H), 6.82 (bs, 1H), 6.25 (bs, 1H), 5.69 – 5.54 (m, 1H), 5.54 – 5.43 (m, 1H), 4.74 – 4.64 (m, 1H), 4.56 (s, 2H), 3.57 – 2.94 (m, 10H), 2.26 – 2.17 (m, 1H), 2.21 (s, 3H), 2.17 – 2.02 (m, 3H), 2.02 – 1.95 (m, 1H), 1.83 – 1.70 (m, 1H), 1.67 – 1.52 (m, 2H), 1.50 – 1.34 (m, 1H), 1.20 – 1.07 (m, 1H).

**^13^C NMR (101 MHz, DMSO-*d*_6_):** δ 163.7, 160.2, 156.4, 147.6, 138.8, 135.0, 133.2, 131.5, 129.0, 95.7, 68.6, 57.9, 50.9, 33.9, 32.2, 29.6, 27.4, 10.8.

**UHPLC**: t*_R_* = 1.51 min, **MS (ESI):** 670 (M + H)^+^.

**Chemical synthesis schemes**

**Scheme 1: Synthesis of 5**

**Scheme 2: Synthesis of 3 and 6**

**Scheme 3: Synthesis of 1 and 4**

**Supplemental note**

**Considerations for optimization of concentration and labeling time for covalent probes.**

In competition binding experiments, increased binding of a test compound to its target protein(s) is determined by the reduction of binding of the target protein(s) to a probe matrix. Resulting IC_50_ values can be strongly influenced by the concentration of the probe matrix, essentially as described by the Cheng-Prusoff relationship ^2^. The influence of the probe matrix can be determined by consecutive affinity enrichments to determine the degree of depletion of proteins on the probe matrix ^2-4^.

For a covalent probe matrix observed IC_50_ values can also be dramatically shifted from the actual half-maximal binding of the inhibitor to its target (K_d_^app^) values if the probe concentration is too high or the reaction time is too long. Unfortunately, the effect of the probe to single proteins cannot be determined with consecutive affinity enrichment experiments when using a covalent probe matrix. However, if the reaction of the probe to its target protein(s) is performed at a time point and a probe concentration at which the probe-enzyme reaction is still in the initial linear phase, the probe concentration does not significantly alter the observed IC_50_ values between tested compound and its target(s) as previously demonstrated by Patricelli et al ^5^. Accordingly, conditions need to be optimized for probe concentration and labeling time to ensure that reaction to targets (in this case, kinases) is in an initial rate phase with subsaturating probe binding. Thus, IC_50_ values that are obtained should closely reflect the binding constants of the tested compound ^6^.

A detailed examination of the kinetics and the influence of a covalent probe on the equilibrium between target and non-covalent inhibitor has been described before ^5^.

**Supporting Figures**

| 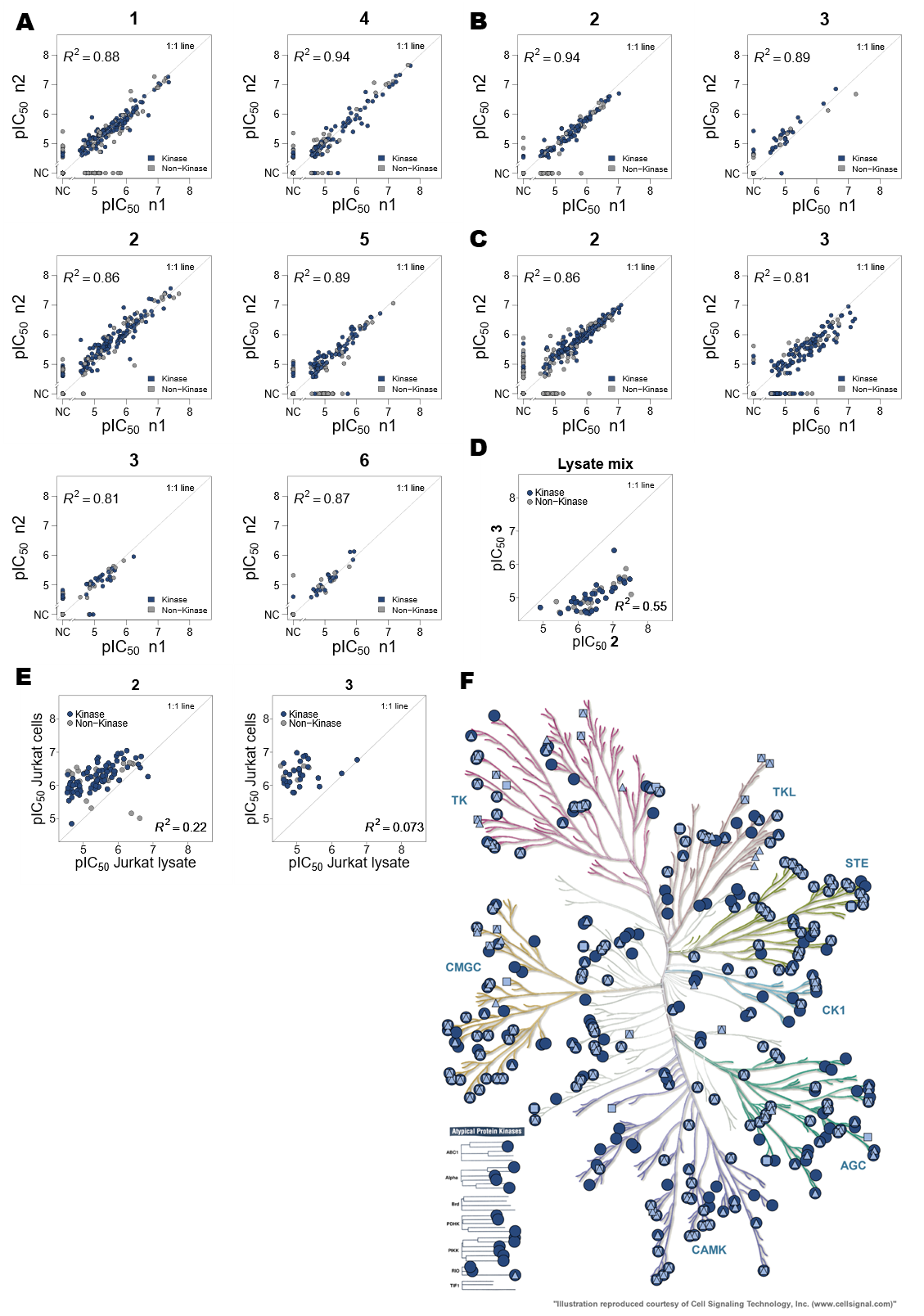 |
| --- |
| Supplemental Figure S1. Characterization of probes with kinobeads.   1. Comparison of binding strengths (pIC_50_ values) determined from kinobeads assay using a mixture of cell extracts (HepG2 Princen, K-562, Hek293, Placenta) for compounds **1** – **6**. Blue dots represent kinases. Grey dots represent non-kinase proteins. 2. Comparison of binding strengths (pIC_50_ values) determined from kinobeads assay using Jurkat cellular extracts for compounds **2** and **3**. Blue dots represent kinases. Grey dots represent non-kinase proteins. 3. Comparison of binding strengths (pIC_50_ values) determined from kinobeads assay using Jurkat cells for compounds **2** and **3**. Blue dots represent kinases. Grey dots represent non-kinase proteins. 4. Comparison of mean binding strengths of competed proteins (>50% competition at 30 µM determined by kinobeads profiling (Fig. S1A)) of compounds **2** and **3** in a mixture of cell extracts. 5. Comparison of mean binding strengths of competed proteins (>50% competition at 30 µM determined by kinobeads profiling (Fig. S1B, C)) of compound **2** or compound **3** in Jurkat cell extracts versus cells. 6. Distribution of protein kinases on the kinome tree of kinases expressed in Jurkat cells (dark blue circles) versus kinases competed with **2** (light blue triangles) or **3** (light blue squares) as evaluated using kinobeads in Jurkat cells. |

| 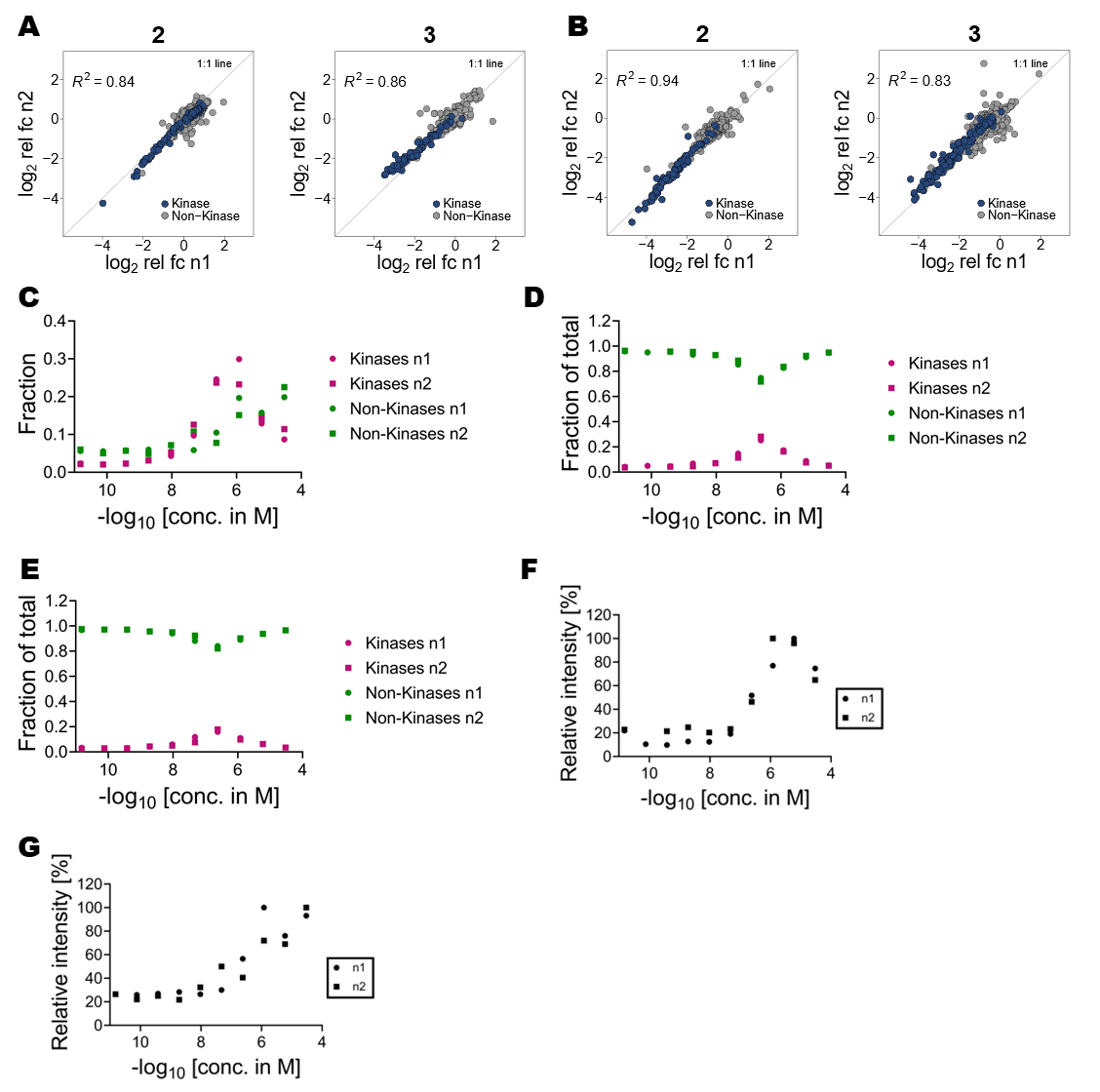 |
| --- |
| Supplemental Figure S2. Characterization of compound **3**.   1. Comparison of log_2_ relative abundance of proteins competed with 10 µM **1** and captured with 1 µM **2** or **3** using Jurkat cellular extracts. Blue dots represent kinases. Grey dots represent non-kinase proteins. 2. Comparison of log_2_ relative abundance of proteins competed with 10 µM **1** and captured with 1 µM **2** or **3** using Jurkat cells. Blue dots represent kinases. Grey dots represent non-kinase proteins. 3. Concentration-dependent capturing of kinases and non-kinases in Jurkat cellular extracts with **3** (n = 2). For each experiment, the cumulative intensity for all kinases or non-kinases for each TMT channel (corresponding to one concentration of **3**) was divided by the total intensity over all channels for all kinases or non-kinases. The sum of all points per experiment per group (kinase or non-kinase) is 1 and the graph displays the relative concentration-dependent behavior of specifically (kinases) and non-specifically (non-kinase) captured proteins by **3**. 4. Concentration-dependent capturing of kinases and non-kinases in Jurkat cellular extracts (n = 2, Fig. S2C). Each data point shows the fraction of the overall intensity within each group per TMT channel (corresponding to one probe concentration). 5. Concentration-dependent capturing of kinases and non-kinases in Jurkat cells (n = 2, Fig. 2F). Each data point shows the fraction of the overall intensity within each group per TMT channel (corresponding to one probe concentration). 6. Summed up intensity of the concentration-dependent capturing of kinases and non-kinases (shown in Fig. 2F, S2E) in Jurkat cells. 7. Summed up intensity of the concentration-dependent capturing of kinases and non-kinases (shown in Fig. S2C, D) in Jurkat cellular extracts. |

| 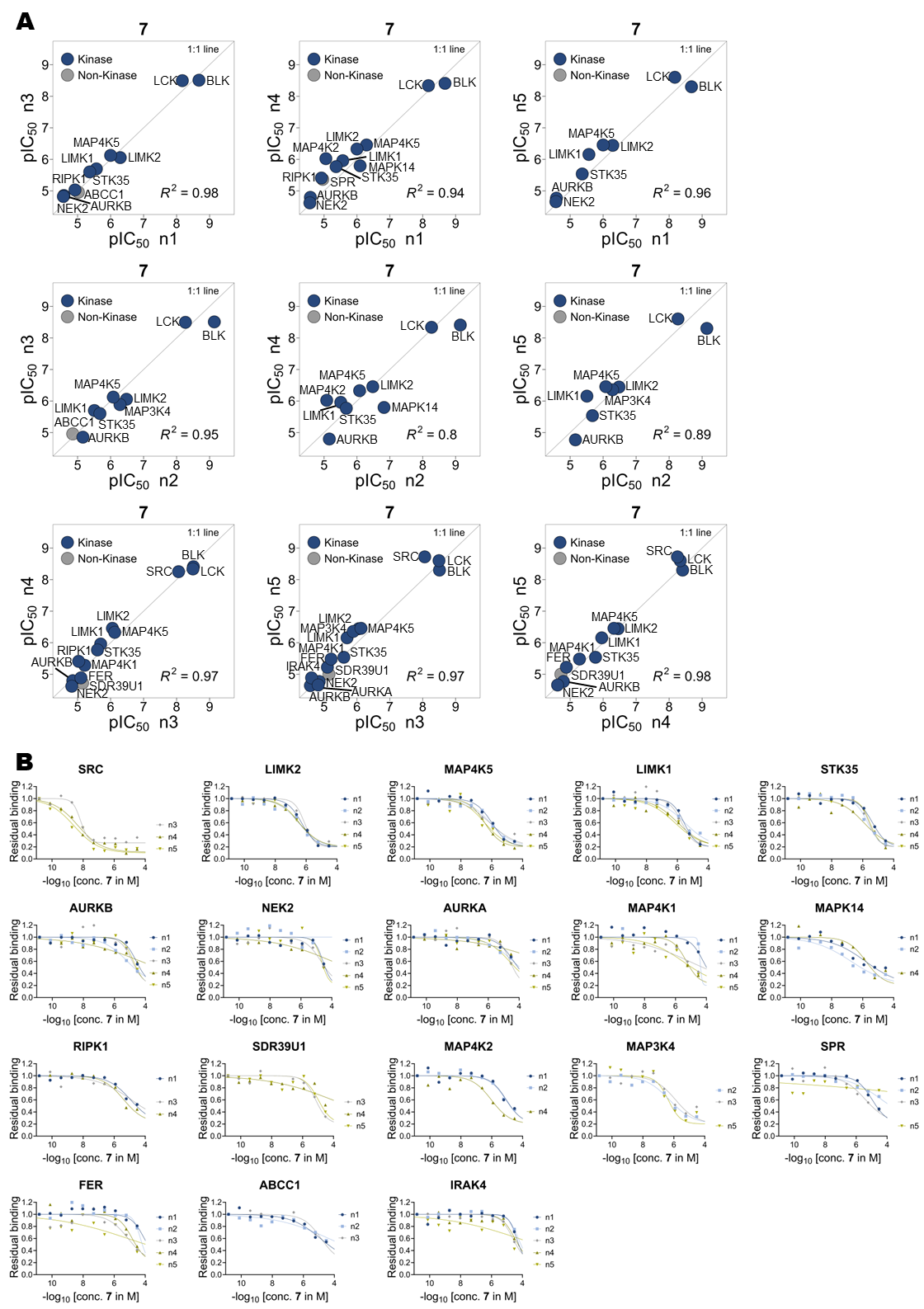 |
| --- |
|  |
| Supplemental Figure S3. Kinase target engagement with Dasatinib in n=5 using compound **3**.   1. Reproducibility of pIC_50_s from target engagement assays in live Jurkat cells testing kinase inhibitor **7**. Comparison of all replicates (n1 / n3; n1 / n4; n1 / n5; n2 / n3; n2 / n4; n2 / n5; n3 / n4; n3 / n5; n4 / n5). Diagonal line represents 1:1 line. R^2^ is derived from a linear fit without predefined intercept. 2. Residual binding curves of competed proteins with valid dose-response curves in at least two out of five replicates using test compound **7** (Dasatinib) (n = 5). pIC_50_ values for each competed protein can be found in Supplemental Table S5.   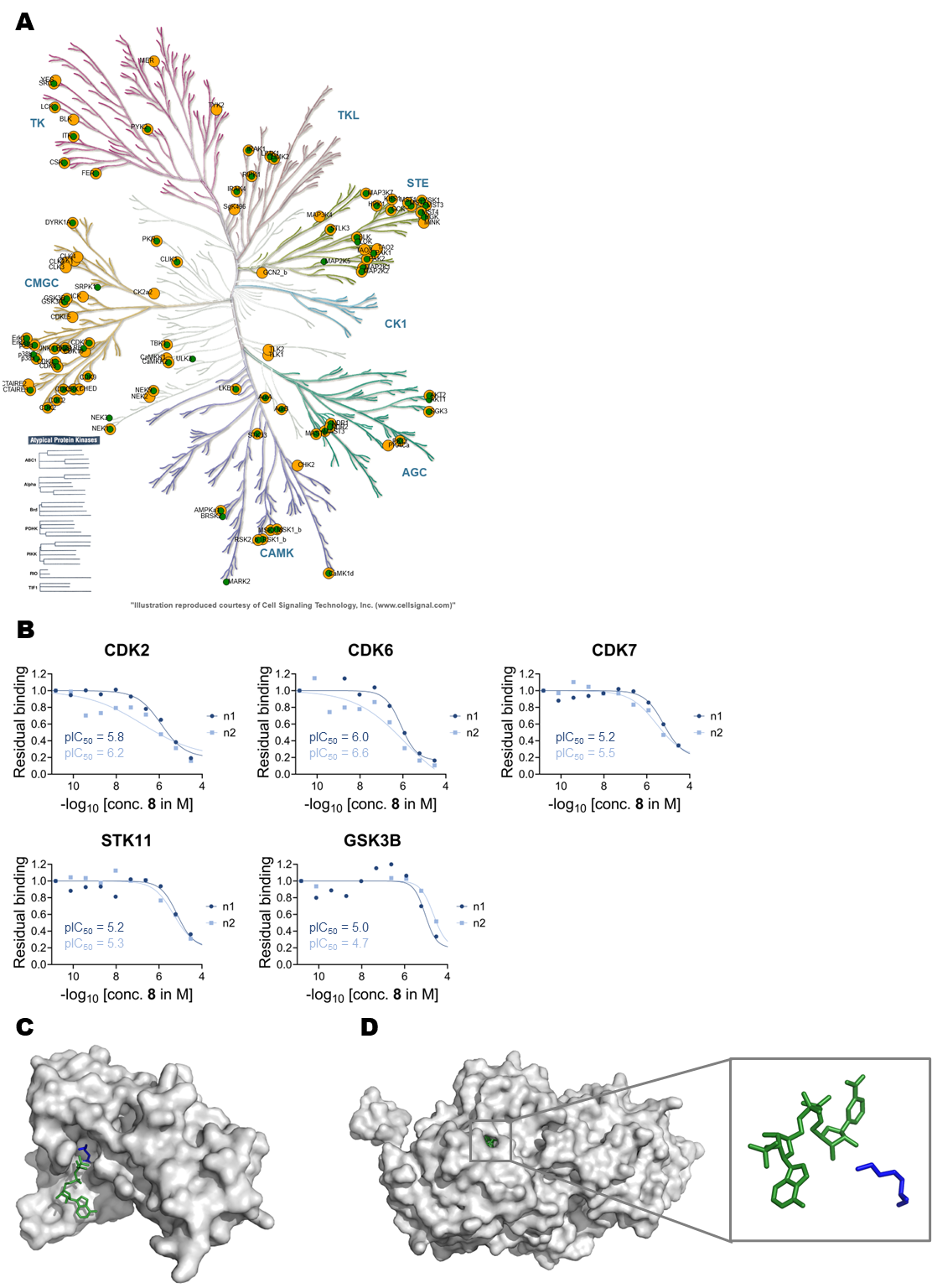 |
| Supplemental Figure S4. Kinome coverage and rationale for capturing of non-kinases.   1. Distribution of the 78 (**2**) and 95 (**3**) competed kinases on the kinome tree. 95 competed kinases by **3** (orange) in comparison to the 78 competed kinases by **2** (green). Like **3**, **2** shows a bias towards the CMGC and STE-branches of the kinome tree and lacks the CK1 branch as well as receptor tyrosine kinases. 68 kinases overlap. 2. Residual binding curves of the competed kinases CDK2, CDK6, CDK7, STK11, and GSK3B using test compound **8** (Dinaciclib) (n = 2). 3. Visualization of hypothesized target lysine in ABCC1. ABCC1 (grey, PDB: 2CBZ) with ATP (green, PDB: 2CBZ). Lys684 is depicted in blue. 4. Visualization of hypothesized target lysine in SPR. SPR (grey, PDB: 6I79) with NADP (green, PDB: 6I79). Lys174 is depicted in blue. |

**Supporting Tables**

Supplementary table S1: Table S1_Kinobeads.xlsx

Proteomics results of kinobeads pulldowns of compounds **1** – **6** from lysate and cells. Refers to Figures 1 C-G & S1 & S4A

Supplementary table S2: Table S2_Jurkat_Expression_proteome_Kinases_KinomeTreeMapping.xlsx

Proteomics results of a Jurkat total lysate. Refers to Figure S1F.

Supplementary table S3: Table S3_Competition.xlsx

Proteomics results of single concentration competition experiments in Jurkat lysate and cells with compound **2** and **3**. Refers to Figures 2B-E & S2A-B.

Supplementary table S4: Table S4_AssayOptimization_Kinase.xlsx

Proteomics results of characterizing compound **3**. Refers to Figure 2 F-I & S2 C-G.

Supplementary table S5: Table S5_TargetEngagement_KinaseInhibitors_OffTargets.xlsx

Proteomics results of the target engagement assay using compound **2** as probe. Refers to Figure 3 C-L & S3 A-B & S4B.

Supplementary table S6: Table S6_Kinobeads_Dasatinib_Dinaciclib.xlsx

Proteomics results of kinobeads pulldowns of **7** + **8**. Refers to Figures 3H+I.

**References**

(1) Zhao, Q.; Ouyang, X.; Wan, X.; Gajiwala, K. S.; Kath, J. C.; Jones, L. H.; Burlingame, A. L.; Taunton, J. Broad-Spectrum Kinase Profiling in Live Cells with Lysine-Targeted Sulfonyl Fluoride Probes. *J Am Chem Soc* **2017**, *139* (2), 680-685. DOI: 10.1021/jacs.6b08536 From NLM.

(2) Sharma, K.; Weber, C.; Bairlein, M.; Greff, Z.; Kéri, G.; Cox, J.; Olsen, J. V.; Daub, H. Proteomics strategy for quantitative protein interaction profiling in cell extracts. *Nature Methods* **2009**, *6* (10), 741-744. DOI: 10.1038/nmeth.1373.

(3) Médard, G.; Pachl, F.; Ruprecht, B.; Klaeger, S.; Heinzlmeir, S.; Helm, D.; Qiao, H.; Ku, X.; Wilhelm, M.; Kuehne, T.; et al. Optimized Chemical Proteomics Assay for Kinase Inhibitor Profiling. *Journal of Proteome Research* **2015**, *14* (3), 1574-1586. DOI: 10.1021/pr5012608.

(4) Eberl, H. C.; Werner, T.; Reinhard, F. B.; Lehmann, S.; Thomson, D.; Chen, P.; Zhang, C.; Rau, C.; Muelbaier, M.; Drewes, G.; et al. Chemical proteomics reveals target selectivity of clinical Jak inhibitors in human primary cells. *Scientific Reports* **2019**, *9* (1), 14159. DOI: 10.1038/s41598-019-50335-5.

(5) Patricelli, M. P.; Nomanbhoy, T. K.; Wu, J.; Brown, H.; Zhou, D.; Zhang, J.; Jagannathan, S.; Aban, A.; Okerberg, E.; Herring, C.; et al. In situ kinase profiling reveals functionally relevant properties of native kinases. *Chem Biol* **2011**, *18* (6), 699-710. DOI: 10.1016/j.chembiol.2011.04.011 From NLM.

(6) Patricelli, M. P.; Szardenings, A. K.; Liyanage, M.; Nomanbhoy, T. K.; Wu, M.; Weissig, H.; Aban, A.; Chun, D.; Tanner, S.; Kozarich, J. W. Functional Interrogation of the Kinome Using Nucleotide Acyl Phosphates. *Biochemistry* **2007**, *46* (2), 350-358. DOI: 10.1021/bi062142x.
